# BCAS1 defines a heterogeneous cell population in diffuse gliomas

**DOI:** 10.18632/oncotarget.28553

**Published:** 2024-01-24

**Authors:** Raquel Morales-Gallel, María José Ulloa-Navas, Patricia García-Tárraga, Ricardo Prat-Acín, Gaspar Reynés, Pedro Pérez-Borredá, Luis Rubio, Vivian Capilla-González, Jaime Ferrer-Lozano, José Manuel García-Verdugo

**Affiliations:** ^1^Laboratory of Comparative Neurobiology, Institute Cavanilles of Biodiversity and Evolutionary Biology, University of Valencia-CIBERNED, Valencia, Spain; ^2^Department of Neurosurgery, Mayo Clinic, Jacksonville, FL 32224, USA; ^3^Department of Neurosurgery, Hospital Universitari i Politècnic La Fe, Valencia, Spain; ^4^Group of Clinical and Translational Research in Cancer, Health Research Institute Hospital Universitari i Politècnic La Fe, Valencia, Spain; ^5^Department of Pathology, Hospital Universitari i Politècnic La Fe, Valencia, Spain; ^6^Department of Integrative Pathophysiology and Therapies, Andalusian Center for Molecular Biology and Regenerative Medicine-CABIMER, Junta de Andalucía-University of Pablo de Olavide-University of Seville-CSIC, Seville, Spain; ^*^These authors contributed equally to this work

**Keywords:** brain tumor, diffuse glioma, oligodendroglioma, glioblastoma, BCAS1

## Abstract

Oligodendrocyte precursor markers have become of great interest to identify new diagnostic and therapeutic targets for diffuse gliomas, since state-of-the-art studies point towards immature oligodendrocytes as a possible source of gliomagenesis. Brain enriched myelin associated protein 1 (BCAS1) is a novel marker of immature oligodendrocytes and was proposed to contribute to tumorigenesis in non-central nervous system tumors. However, BCAS1 role in diffuse glioma is still underexplored. This study analyzes the expression of BCAS1 in different tumor samples from patients with diffuse gliomas (17 oligodendrogliomas; 8 astrocytomas; 60 glioblastomas) and uncovers the molecular and ultrastructural features of BCAS1^+^ cells by immunostaining and electron microscopy. Our results show that BCAS1^+^ cells exhibit stellate or spherical morphology with similar ultrastructural features. Stellate and spherical cells were detected as isolated cells in all studied gliomas. Nevertheless, only stellate cells were found to be proliferative and formed tightly packed nodules with a highly proliferative rate in oligodendrogliomas. Our findings provide a comprehensive characterization of the BCAS1^+^ cell population within diffuse gliomas. The observed proliferative capacity and distribution of BCAS1^+^ stellate cells, particularly in oligodendrogliomas, highlight BCAS1 as an interesting marker, warranting further investigation into its role in tumor malignancy.

## INTRODUCTION

Diffuse gliomas are tumors derived from glial cells. They are characterized by an extensive, infiltrative growth into the dense network of neuronal and glial processes of the central nervous system (CNS) [1]. These tumors are the most common primary malignant brain tumors in adult population. Diffuse gliomas are subclassified into three tumor subtypes: oligodendroglioma (OG), astrocytoma (AS) and glioblastoma (GB) [2]. Although the incidence rate differs between them (GB 9.23 per 100 000, AS 4.68 per 100 000, and OG 3.57 per 100 000), the three tumor subtypes are associated with a significant mortality and morbidity [3, 4].

The classification of each subtype of diffuse glioma is based on histopathological and molecular analysis, including mutations in isocitrate dehydrogenase (IDH) and alpha thalassemia/mental retardation syndrome X-linked (ATRX). The World Health Organization (WHO) has assigned the malignancy grade (grade 2, 3 or 4) according to the mitotic activity, necrosis, and microvascular proliferation [2, 5, 6]. Despite the enormous progress in molecular characterization, specific treatments specially for patients with high-grade tumors are non-existent. Standard of care for glioma patients consist of surgery, followed by temozolomide chemotherapy and radiotherapy [7]. Finding molecules that define proliferating cell populations in tumors will lead to a better understanding of the molecular mechanisms of tumorigenesis, the discovery of new prognostic biomarkers and the identification of novel targets that will help to promote an earlier and more refined diagnosis and treatment.

A recent multi-omics study has subclassified a heterogeneous group of gliomas into different clusters in accordance with the transcriptional profile of the neoplastic cells [8]. Among these classifications, one subgroup showed to be highly similar to oligodendrocyte precursor cells (OPCs). Interestingly, OPC-like transcriptional profile is associated with greater malignancy tumors, while mature neuronal or oligodendrocytic profile appear to be less aggressive [8]. For this reason, studying OPC markers is of great interest. In the last years, brain enriched myelin associated protein 1 (BCAS1), also known as breast carcinoma amplified sequence 1 or novel amplified in breast cancer 1, is a protein that has been stablished as a marker of early myelinating oligodendrocytes in the mouse and human brain [9]. The expression of this protein has been also found in neoplastic cells from non-CNS tissues, such as breast [10], prostate [11], pancreatic [12], colorectal [13] and gastric cancer [14]. Recently, our research group analyzed the expression of BCAS1 in a patient with grade 2 OG. In this case report, a compact cluster of BCAS1^+^ cells was described for the first time in a glioma [15]. Finally, a novel splice variant of BCAS1 has been linked with high proliferation and migration rate of the glioblastoma [16]. Given all the evidence showing that this OPC marker might play an important role in gliomagenesis, in this study, we analyzed the expression of BCAS1 in a cohort of 85 samples from patients diagnosed with diffuse gliomas, including OG, AS and GB. We examined the ultrastructural features of BCAS1^+^ cells their molecular characteristics and the microenvironment of these cells. Our study provides the first evidence of BCAS1 expression in diffuse gliomas, with a heterogeneous pattern of distribution across all tumor subtypes. BCAS1-expressing cells display two different morphologies. Both morphologies exhibit similar ultrastructural features, but differ in spatial organization and proliferation capacity, suggesting that they correspond to the same cell population but in different activation state.

## RESULTS

### BCAS1 is expressed in all diffuse gliomas and correlates with two different cell morphologies

**Figure 1:**
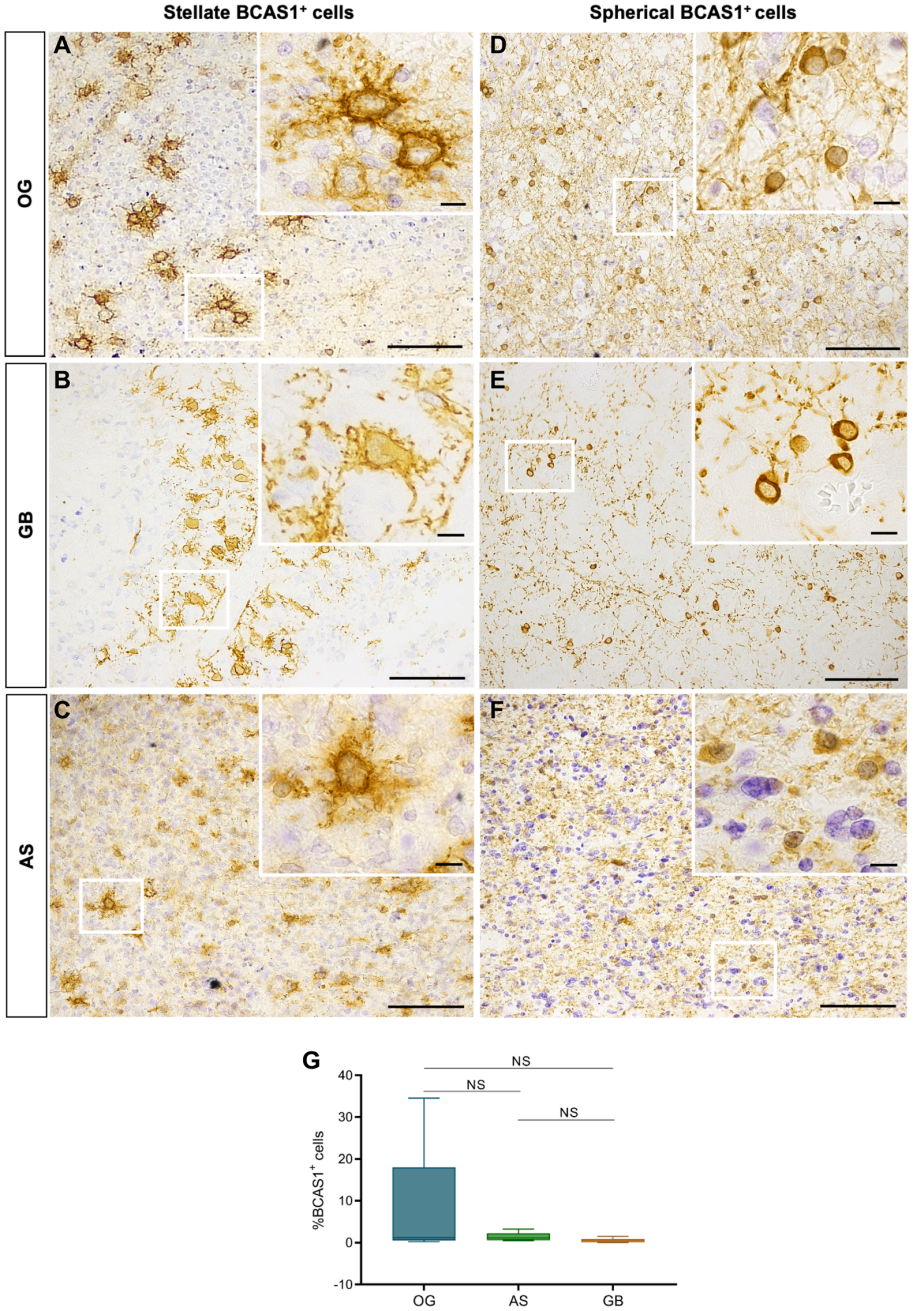
Diffuse gliomas BCAS1-expressing cells have two different morphologies. (**A**) In OG, BCAS1 immunohistochemical staining shows stellate BCAS1^+^ cells individually distributed or forming small groups of 2 to 5 cells within the tissue. The magnification shows a detail of a BCAS1^+^ cell with stellate morphology in OG. (**B**) BCAS1 immunohistochemical staining and magnification showing BCAS1^+^ cells with stellate morphology in GB. (**C**) BCAS1 immunohistochemical staining in AS presents a randomly distributed stellate BCAS1^+^ cells within the tumor. The magnification shows a detail of a BCAS1^+^ cell with stellate morphology in AS. (**D**) In OG, also spherical BCAS1-expressing cells were detected. The magnification shows a detail of a BCAS1^+^ cell with spherical morphology in OG. (**E**) Randomly distributed spherical BCAS1^+^ cells are present in GB. (**F**) BCAS1 immunohistochemical staining also shows BCAS1^+^ cells with spherical morphology in AS. In all images, nuclei were counterstained with cresyl violet (purple). For DAB immunohistochemical staining: *n* = 17 OG, *n* = 8 AS, *n* = 60 GB. Scale bars: A–F) 100 μm; inserts, 10 μm. (**G**) Quantification of the percentage of total BCAS1^+^ cells in OG (*n* = 12), AS (*n* = 8) and GB (*n* = 10). Kruskal–Wallis test w/Dunn’s multiple comparisons. Center lines in the boxes indicate the median and whiskers indicate the minimum and the maximum values. Abbreviation: NS: not significant.

To examine the expression of BCAS1 in a representative cohort of diffuse gliomas, we collected samples from the tumor core of three diffuse glioma subtypes (i.e., 17 OG, 8 AS and 60 GB from different patients). After the samples underwent pathological examination and subsequent classification at the Hospital Universitari i Politècnic la Fe of Valencia (Spain), we performed immunohistochemistry to detect the expression of BCAS1. The results revealed BCAS1 expression in all OG and AS samples and in the 90% of the GB samples (Figure 1A–1F). After quantifying the number of BCAS1^+^ cells in the hot spots of each tumor (i.e., the area with more BCAS1^+^ cells in the tissue section), we did not find statistically significant differences in the number of BCAS1^+^ cells between OG, AS and GB (OG 10.44 ± 4.78%; AS 1.37 ± 0.34%; GB 0.58 ± 0.14%; *p*-value = 0.0786 (Figure 1G and Supplementary Table 1). In addition, we compared the percentage of BCAS1^+^ cells with other clinical data, as the gender, the age of the patients, their progression-free survival and overall survival values, but not statistically differences were observed (see Supplementary Figure 1).

**Figure 2:**
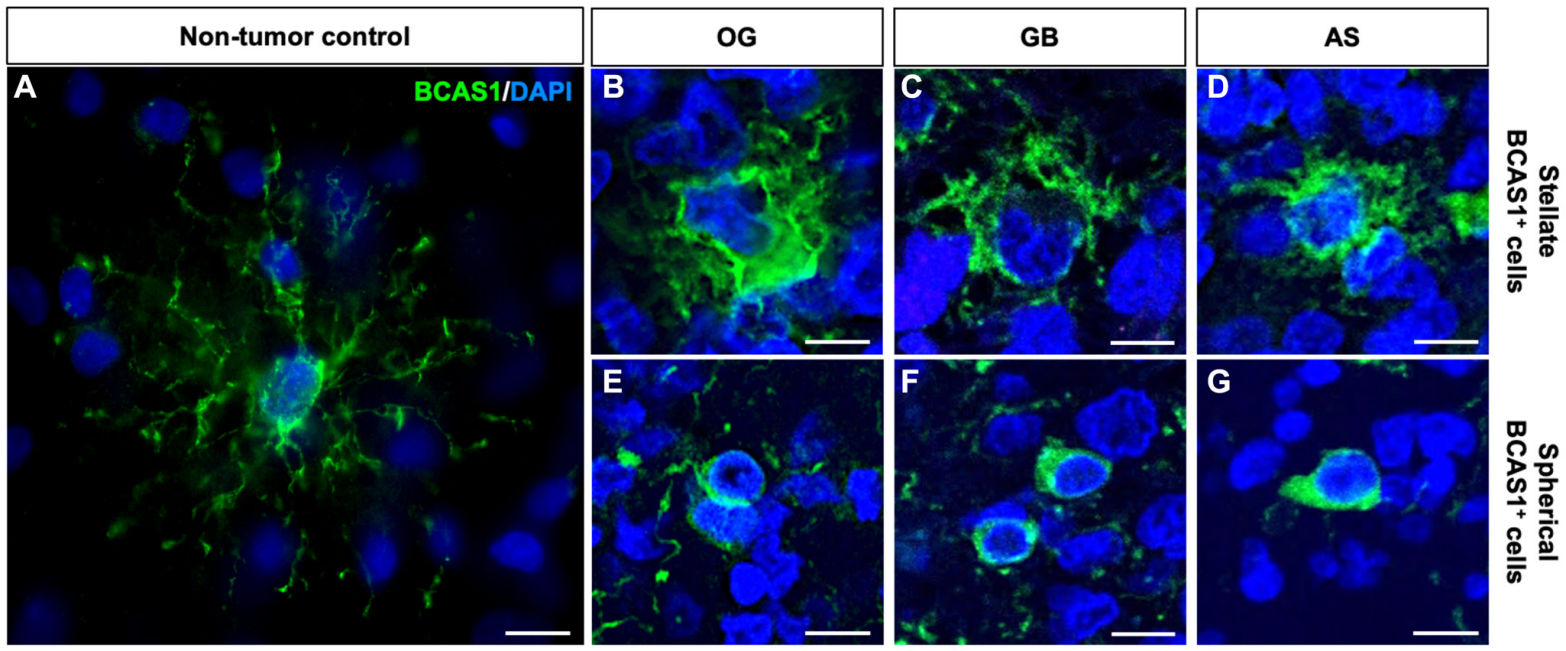
BCAS1^+^ cells in diffuse gliomas are morphologically different from BCAS1^+^ cells in healthy brain tissue. (**A**) Detail of a BCAS1 immunofluorescence staining in a non-tumor control sample reveals BCAS1-expressing cell (green) presents small nucleus and reduced cytoplasm with numerous, thin and branched prolongations. In all images, nuclei were counterstained with DAPI (blue). (**B**) BCAS1 immunofluorescence staining showing a BCAS1^+^ cell (green) with stellate morphology in OG. (**C**) BCAS1 immunofluorescence staining showing a BCAS1^+^ cell (green) with stellate morphology in GB. (**D**) BCAS1 immunofluorescence staining showing a BCAS1^+^ cell (green) with stellate morphology in AS. (**E**) BCAS1 immunofluorescence staining showing a BCAS1^+^ cell (green) with spherical morphology in OG. (**F**) BCAS1 immunofluorescence staining showing a BCAS1^+^ cell (green) with spherical morphology in GB. (**G**) BCAS1 immunofluorescence staining showing a BCAS1^+^ cell (green) with spherical morphology in AS. Scale bars: 10 μm.

After detecting BCAS1-positive cells in most diffuse gliomas, we focused our efforts on further characterizing these cells. The immunohistochemistry revealed that BCAS1-expressing cells present two different morphologies: stellate or spherical (Figure 1A–1F). We defined stellate BCAS1^+^ cells as those cells showing numerous prolongations (star-like shape), large size, abundant cytoplasm, irregular shaped nucleus, and high BCAS1 immunoreactivity in the plasma membrane (Figure 1A–1C). In contrast, spherical BCAS1^+^ cells were smaller, with none or few thin prolongations and reduced cytoplasm that exhibited high BCAS1 immunoreactivity (Figure 1D–1F). Importantly, stellate and spherical BCAS1-expressing cells were not observed in non-tumor control samples (i.e., non-affected white matter samples from resections of focal cortical dysplasia in 3 different patients). In contrast to tumor BCAS1^+^ cells, non-tumor BCAS1^+^ cells exhibited a thinner layer of cytoplasm surrounding a smaller round nucleus and numerous highly branched processes (Figure 2A and Supplementary Figure 2). These observations are in line with the morphology previously described for BCAS1^+^ oligodendrocytes in human and mouse brain tissue [9, 17]. Stellate and spherical BCAS1^+^ cells were detected in the three tumor subtypes analyzed (Figure 2B–2G). Although in most cases the same sample presented both cell morphologies, in some tumors only spherical BCAS1^+^ cells (2 OG, 2 AS and 24 GB), or only stellate BCAS1^+^ cells (2 OG and 3 GB) could be detected. We hypothesize that both BCAS1^+^ cell morphologies coexist in all tumors, but the limited area that we examined may influence the results.

### Stellate and spherical BCAS1^+^ cells present similar ultrastructural and molecular features

**Figure 3:**
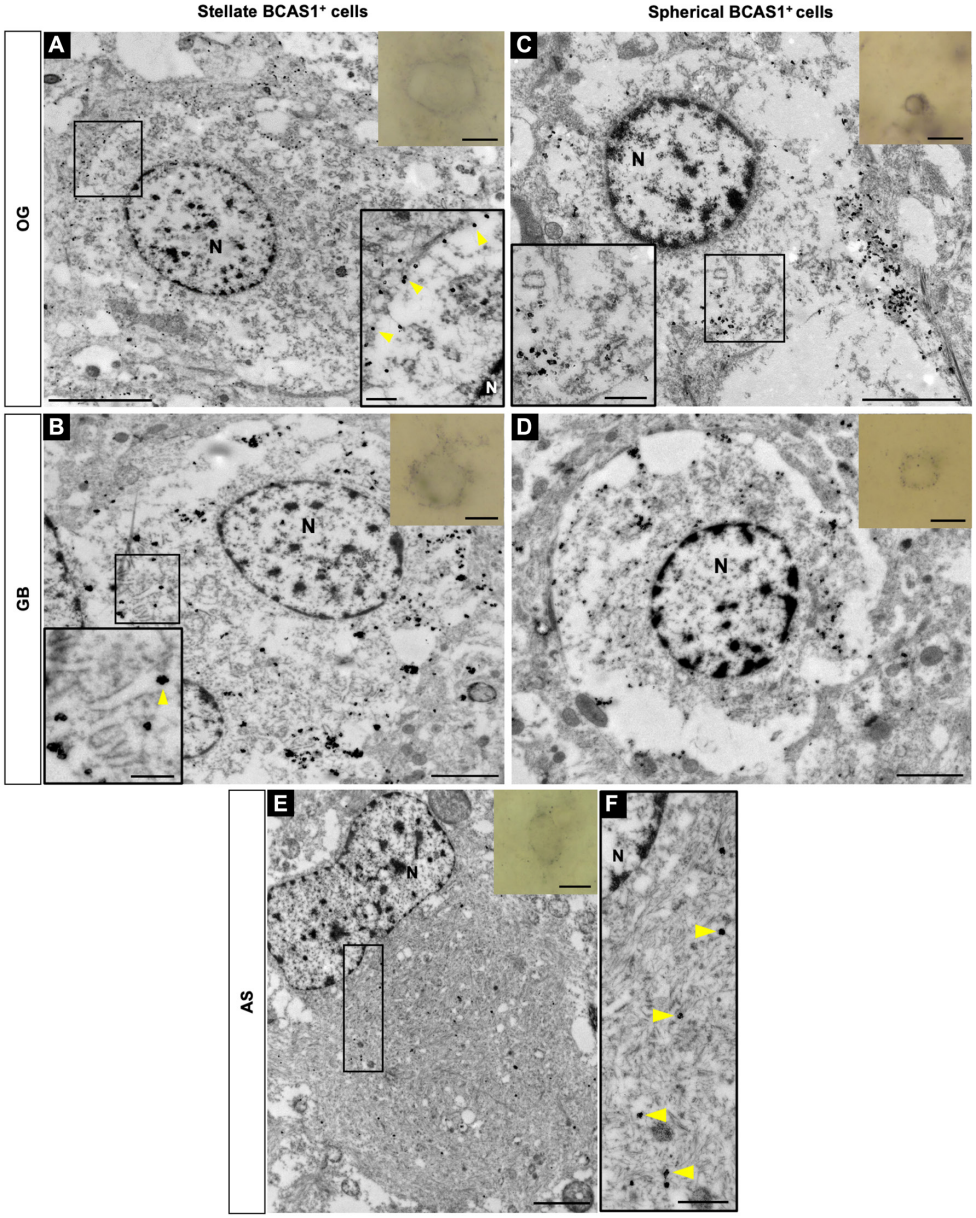
Subcellular localization of BCAS1 protein and ultrastructural features of BCAS1^+^ cells. Transmission electron microscopy images of immuno-gold BCAS1-positive cells. Inserts in the right top corner are the optical microscope vision of the cells. (**A**) In OG, stellate cells are large, present abundant cytoplasm and loose chromatin. Magnification shows a detail of BCAS1 label in the plasma membrane (yellow arrowheads) and in close association with the abundant, short and dilated RER. (**B**) In GB, stellate cells share ultrastructural characteristics with those in OG. Magnification shows a detail of the BCAS1 label in RER (yellow arrowheads). (**C**) In OG, spherical cells have a small and round nucleus with a more condensed chromatin and present scarce cytoplasm. The subcellular BCAS1 location was also in the plasma membrane and in the RER. Magnification shows a detail of the BCAS1 label also in RER (yellow arrowheads). (**D**) In GB, spherical cells share ultrastructural characteristics with those in OG. (**E**) In AS, BCAS1-expressing cells present an irregular nucleus and an extensive cytoplasm with abundant intermediate filaments. (**F**) Magnification of the cytoplasm of a BCAS1^+^ cell in AS showing the subcellular location of BCAS1 label in the intermediate filaments (yellow arrowheads). *n* = 2 OG, *n* = 2 AS, *n* = 2 GB. N: nucleus. Scale bars: optical microscope vision) 5 μm; A, insert) 5 μm, 2 μm; B, insert) 2 μm, 500 nm; C, insert) 2 μm, 500 nm; D) 2 μm; E) 2 μm; F) 500 nm.

The presence of two morphologically different BCAS1^+^ cells leads to the question of whether they correspond to two different cell types, or, on the contrary, both cellular morphologies belong to the same cell type, but in different activation state or cell-cycle stage. To better understand the differences between these two cellular morphologies, we performed immunogold labeling against BCAS1 to study their ultrastructural features by transmission electron microscopy (Figure 3). BCAS1^+^ cells with stellate or spherical morphology were selected from semithin sections by light microscope.

In OG and GB, our results showed that BCAS1 is mostly expressed in the plasma membrane and, in some cases, is associated with the rough endoplasmic reticulum (RER) in both stellate and spherical cell types (Figure 3A–3D). Interestingly, stellate BCAS1 cells present abundant, short and dilated RER throughout the cytoplasm that resembles an ultrastructural hallmark of oligodendrocytes (Figure 3E, 3F). In addition, ultrastructural analysis also suggests that both morphologies have different activation state based on chromatin compaction levels. While stellate cells show loose chromatin, similar to active cells (Figure 3A, 3B), spherical cells display a higher degree of compaction, resembling quiescent cells (Figure 3C, 3D). In the case of AS, all BCAS1^+^ cells studied by transmission electron microscopy showed similar features, making difficult to differentiate stellate and spherical morphologies. BCAS1 expression was restricted to the cytoplasm, which was extensive and had a nucleus with invaginations and chromatin organized in small granules. However, the main characteristic for BCAS1^+^ cells in AS was the presence of abundant intermediate filaments that correlated with astrocytic phenotype. BCAS1 label was associated with these intermediate filaments (Figure 3E, 3F). Therefore, the ultrastructure of BCAS1^+^ cells in AS differed from that OG and GB.

**Figure 4:**
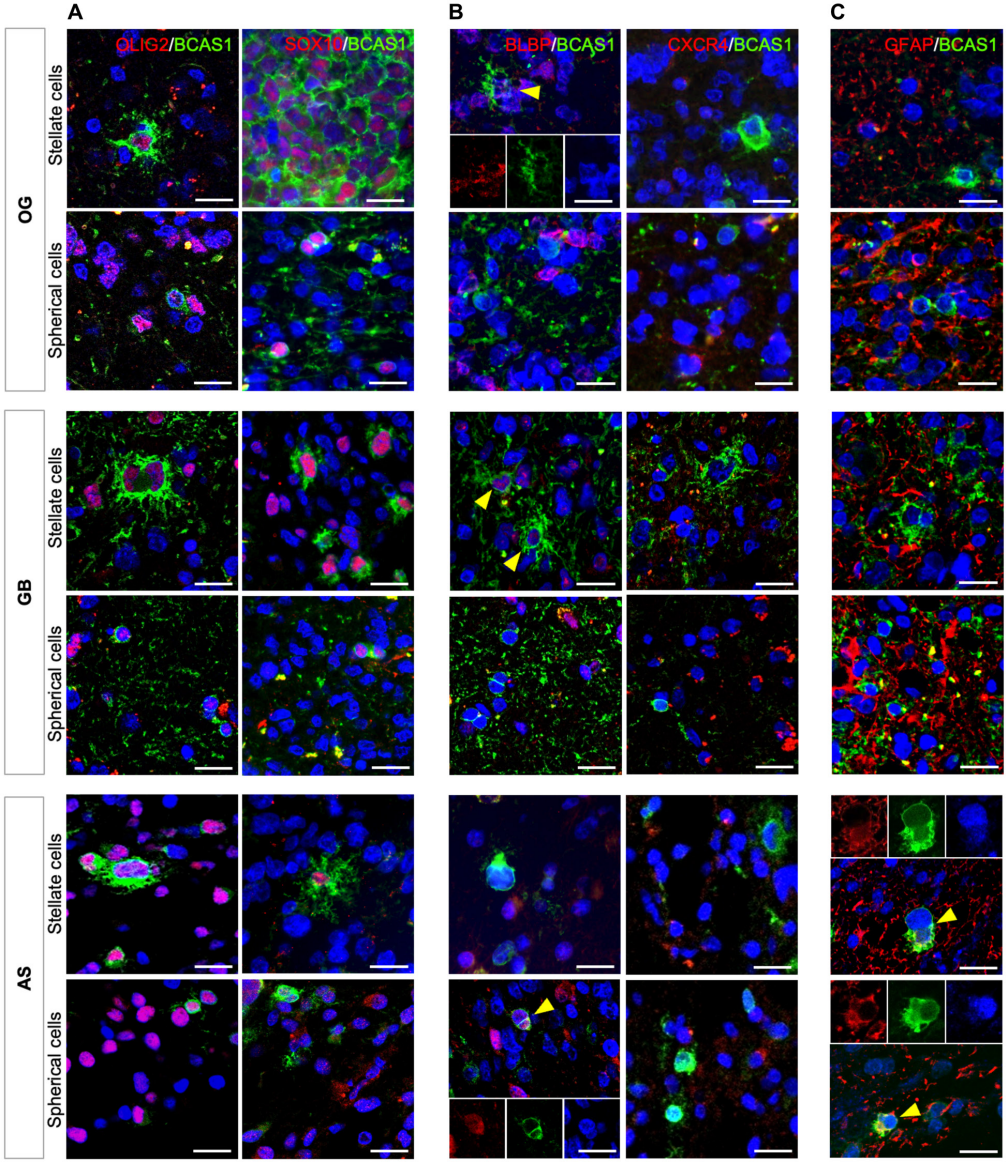
BCAS1^+^ cells share molecular characteristics in diffuse gliomas. Immunofluorescence images of stellate and spherical BCAS1^+^ cells in the different types of diffuse gliomas studied. (**A**) OLIG2/BCAS1 and SOX10/BCAS1 double immunofluorescences showing that, in all tumors, BCAS1-expressing cells, regardless its morphology, co-express OLIG2, but not all BCAS1^+^ cells co-express SOX10. (**B**) BLBP/BCAS1 and CXCR4/BCAS1 double immunofluorescences reveals some BCAS1^+^ cells co-localization for BCAS1^+^ cells with BLBP (yellow arrowheads, three channels insert), but any BCAS1-expressing cell was co-label with CXCR4 marker. (**C**) GFAP/BCAS1 double immunofluorescence. Only in AS, BCAS1^+^/GFAP^+^ cells were detected (yellow arrowheads). For each double immunofluorescence staining: *n* = 1 OG, *n* = 1 AS and *n* = 1 GB. Scale bars: 20 μm.

Since BCAS1 has been studied as a marker of a very defined stage of differentiation in oligodendrocytes [9], we decided to study a set of other oligodendrocytic markers (i.e., OLIG2 and SOX10) for different stages of differentiation to compare the neoplastic population to the non-neoplastic oligodendrocyte population. Furthermore, we also studied markers related to glial cell proliferation (i.e., CXCR4 and BLBP) [18–21] and the astrocyte marker glial fibrillary acidic protein (GFAP) [22] (see Supplementary Table 2). Immunofluorescence analysis suggested that all BCAS1^+^ cells, regardless of their morphology, co-expressed OLIG2, but not all of them co-localized with SOX10 (Figure 4A). We did not detect BCAS1 and CXCR4 co-localization, but some BCAS1^+^ cells were co-stained with BLBP marker (stellate cells in OG and GB, and spherical cells in AS) (Figure 4B). This confirmed that BCAS1^+^ cells display similar characteristics to pre-myelinating oligodendrocytes [9, 23]. In addition, co-expression of GFAP and BCAS1 was exclusively found in AS samples, supporting the ultrastructural observation of highly filamentous BCAS1-expressing cells (Figure 4C).

### Stellate BCAS1^+^ cells can form tightly packed nodules in OG

**Figure 5:**
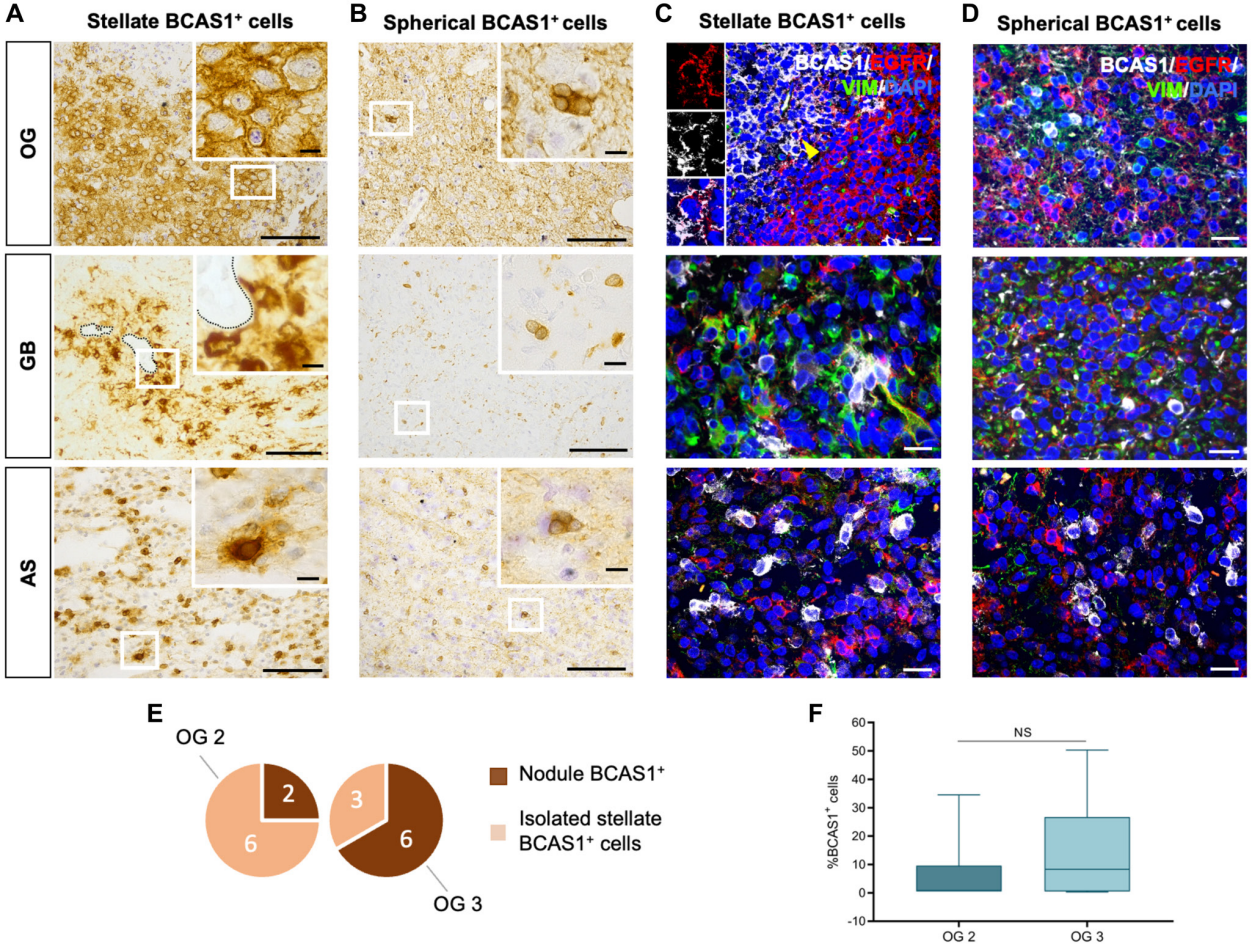
BCAS1^+^ cells in OG form nodules that are surrounded by EGFR^+^ cells. (**A**) DAB immunohistochemical staining reveals stellate BCAS1-expressing cells are distributed forming compacted packages, called BCAS1^+^ nodules, only in OG. In GB, stellate BCAS1^+^ cells are aggregated close to blood vessels (marked by a dotted line). In AS, stellate BCAS1^+^ cells are individualized within the tumor tissue. (**B**) DAB immunohistochemical staining shows spherical BCAS1-expressing cells are individualized or forming groups of 2 to 5 cells in the three tumors studied. (**C**) Triple immunofluorescence showing the microenvironment of stellate BCAS1^+^ cells. In OG, BCAS1^+^ nodule (white) is circled by EGFR-expressing cells (red). Cells co-expressing BCAS1 and EGFR (yellow arrowhead) were observed in the periphery of BCAS1^+^ nodules in OG. The insert highlights a BCAS1^+^/EGFR^+^ cell. In GB and AS, stellate BCAS1^+^ cells are intermingled with the other cell population studied (EGFR^+^, VIM^+^ and EGFR^+^/VIM^+^). (**D**) Triple immunofluorescence showing the microenvironment of spherical BCAS1^+^ cells. In all tumors studied, spherical BCAS1^+^ cells are intermixed with the other cell population analyzed (EGFR^+^, VIM^+^ and EGFR^+^/VIM^+^). (**E**) Number of OG studied in which BCAS1^+^ nodules or only isolated BCAS1^+^ cells have been detected, regarding its malignancy grade (2 or 3). In more malignant OG (grade 3) samples, BCAS1^+^ nodules were observed. (**F**) Quantification of the percentage of total BCAS1^+^ cells in OG with different malignancy grade (OG 2 or OG 3). *n* = 6 grade 2 OG, *n* = 6 grade 3 OG; Mann–Whitney test. Center lines in the boxes indicate the median and whiskers indicate the minimum and the maximum values. Abbreviation: NS: not significant. For DAB immunohistochemical staining: *n* = 17 OG, *n* = 8 AS, *n* = 60 GB. For triple immunofluorescence staining: *n* = 11 OG, *n* = 3 AS, *n* = 16 GB. Scale bars: A, B) 100 μm (inserts: 10 μm); C, D) 20 μm.

To examine the spatial distribution of stellate and spherical BCAS1^+^ in OG, AS and GB, we further analyzed the immunohistochemically stained sections (Figures 1, 5). The majority of stellate and spherical cells do not form groups and are randomly distributed (Figure 1). Intriguingly, only in OG, we detected very compact clusters of stellate BCAS1^+^ cells forming tightly packed nodule-like structures, that we called BCAS1^+^ nodules (Figure 5A). In addition, BCAS1^+^ cells in GB tend to cluster close to the blood vessels (Figure 5A and see Supplementary Figure 3). Regarding the spherical BCAS1^+^ cells, instead of forming compacted nodules, we only detected small groups of 2 to 5 cells in all subtypes of diffuse gliomas studied (Figure 5B).

Then, we performed immunofluorescence against BCAS1, EGFR and VIM to study the microenvironment around BCAS1^+^ cells. EGFR and VIM are markers previously associated with proliferative glioma cells [24–27]. Our results indicated that, generally, BCAS1-expressing cells do not co-localize with EGFR or VIM. When BCAS1^+^ cells appear isolated, regardless its morphology, they are frequently intermingled between EGFR^+^, VIM^+^ or EGFR^+^/VIM^+^ cells, not following any pattern of distribution (Figure 5C, 5D). Interestingly, BCAS1^+^ nodules were surrounded by a halo-like structure of EGFR^+^ cells. While most BCAS1-expressing cells do not co-localize with EGFR, BCAS1^+^/EGFR^+^ cells were found in the periphery of BCAS1^+^ nodules in OG (Figure 5C-insert).

Intriguingly, when we evaluated the density of BCAS1^+^ cells in OG (quantification of the total number of BCAS1^+^ cells in tumor hot spots), our results depicted that grade 3 OG show a higher number of BCAS1^+^ nodules than grade 2 OG. This result was in concordance with an increased number of BCAS1^+^ cells found in grade 3 OG, as compared to grade 2 OG, although the result is not statistically significant (OG 2 6.44 ± 5.62%; OG 3 14.45 ± 7.90%; *p*-value = 0.5887) (Figure 5E, 5F; see Supplementary Table 1).

The capacity of the stellate BCAS1^+^ cells of clustering in nodules in OG, together with the increased number of BCAS1^+^ nodules found in more malignant OG, leads to the question of: what is the function of these clusters within the tumor?

### BCAS1^+^ nodules present a high proliferative capacity

**Figure 6:**
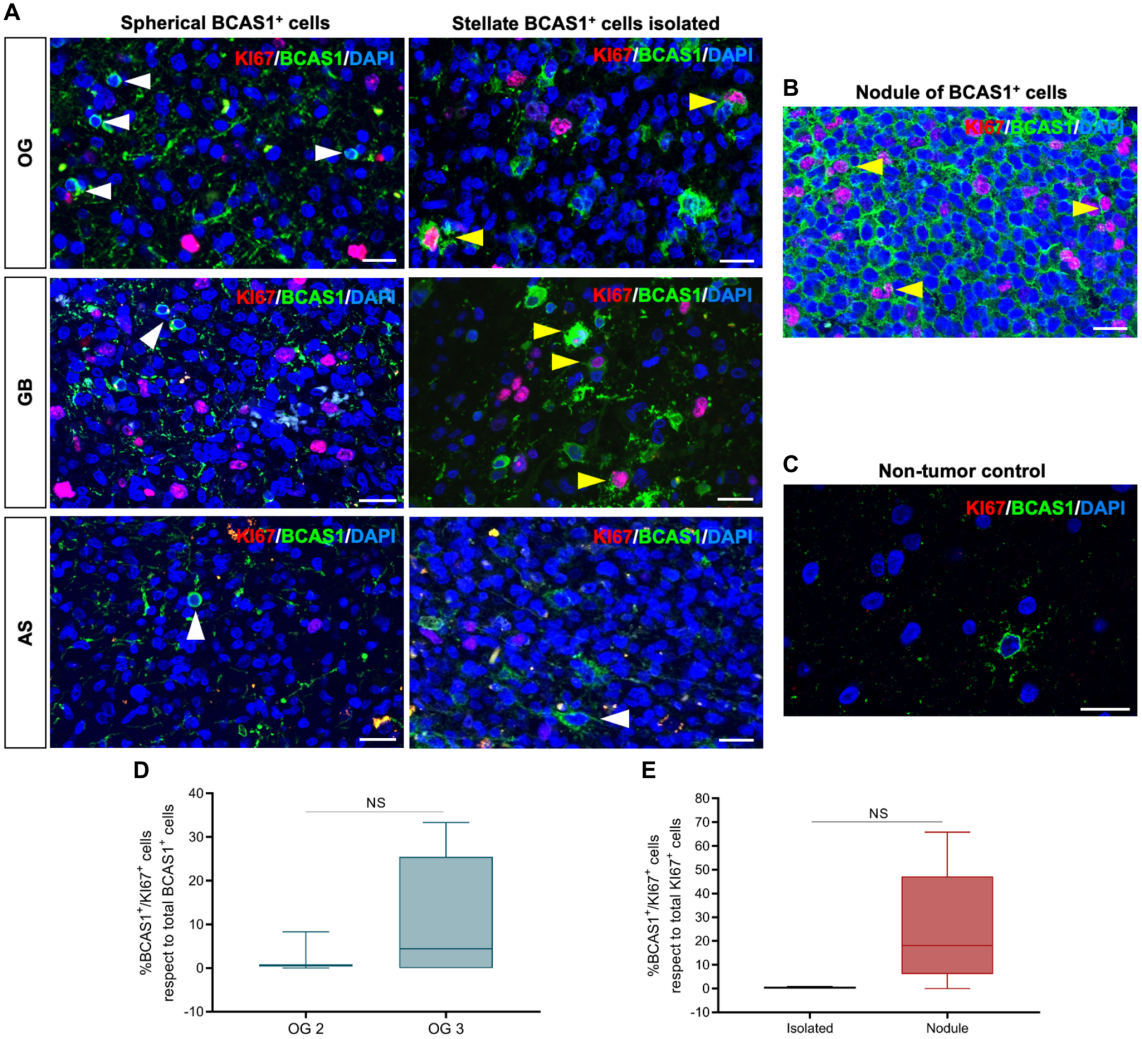
Stellate BCAS1^+^ cells possess proliferate capacity. (**A**) BCAS1 and KI67 double immunofluorescence reveal the capacity to proliferate of stellate BCAS1^+^ cells in OG and GB (yellow arrowheads). In AS, stellate BCAS1^+^ cells do not proliferate (white arrowhead). Spherical BCAS1^+^ cells do not proliferate in any diffuse glioma analyzed (white arrowheads). (**B**) BCAS1^+^ cells forming nodules also display a proliferative capacity (yellow arrowheads). (**C**) BCAS1^+^ cells do not proliferate in non-tumor brain tissue. (**D**) Proliferation rate of the BCAS1^+^ in grade 2 and grade 3 OG. (**E**) Proliferation rate of the BCAS1^+^ cells when comparing the isolated and nodule distribution pattern. For double immunofluorescence staining: *n* = 17 OG, *n* = 8 AS, *n* = 60 GB. For (d) quantification: *n* = 3 grade 2 OG, *n* = 6 grade 3 OG; Mann–Whitney test. Center lines in the boxes indicate the median and whiskers indicate the minimum and the maximum values. Abbreviation: NS: not significant. For (e) quantification: *n* = 4 OG with isolated BCAS1^+^ cells, *n* = 5 OG with BCAS1^+^ nodules; Mann–Whitney test. Center lines in the boxes indicate the median and whiskers indicate the minimum and the maximum values. Abbreviation: NS: not significant. Scale bars: A, B) 25 μm; C) 20 μm.

Given that BCAS1^+^ nodules were structures that we frequently found among grade 3 OG, we wanted to explore the proliferative capacity of the cells that comprise the nodules to better understand the formation of these compact clusters. Therefore, we performed a double immunostaining against BCAS1 and KI67 in representative areas of the tumors (areas containing spherical cells, ungrouped stellate cells and BCAS1^+^ nodules) (Figure 6). The results showed that only stellate BCAS1^+^ cells proliferate, regardless of being isolated or forming nodules. These proliferative BCAS1^+^ cells were found in OG and GB, but they were absent in AS (Figure 6A, 6B). Furthermore, proliferating BCAS1^+^ cells were not observed in non-tumor control sample, supporting previous studies in mouse and human tissue [9] (Figure 6C). This suggests that BCAS1^+^ cells detected in diffuse gliomas could correspond to two cell types of the same cell population in different activation state. The fact that we could not detect proliferating BCAS1 positive cells in AS corroborates that BCAS1^+^ cells in AS might differ in nature from BCAS1^+^ cells in OG and GB. This result is in line with what we previously observed in ultrastructural and molecular studies.

Finally, we further analyzed the proliferative capacity of the stellate BCAS1^+^ cells. We quantified the number of stellate BCAS1^+^ proliferating cells in OG for statistical comparison according to malignancy grade (Figure 6D). Even though there was no significant difference, a trend pointing towards a higher proliferative capacity of BCAS1^+^ cells in grade 3 OG was observed (2.98 ± 2.68% OG 2; 10.84 ± 5.74% OG 3; *p*-value: 0.6936). In addition, BCAS1^+^ cells within the nodules exhibited a proliferation rate of 25%, while only 0.38% of isolated BCAS1^+^ cells proliferated. However, the difference was not statistically significant (0.38 ± 0.23% isolated; 24.94 ± 11.20% nodule; *p*-value: 0.1054) (Figure 6E). This data suggested that BCAS1^+^ nodules could be hot spots for proliferation contributing to tumor growth.

### Diffuse gliomas present an aneuploidy of chromosome 20 with undetected amplification of the *BCAS1* gene

**Figure 7:**
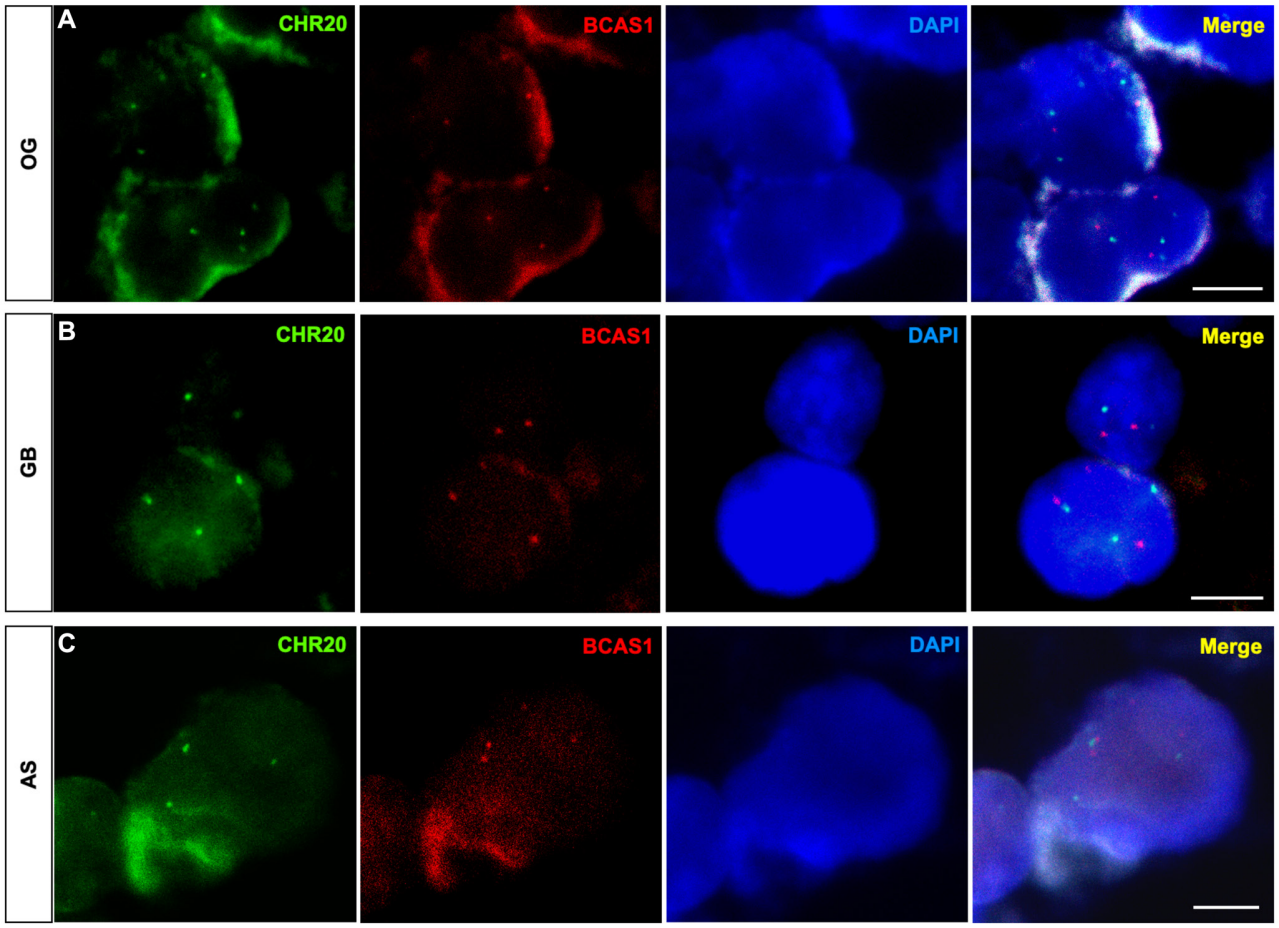
Diffuse glioma cells exhibit numerical aberrations of chromosome 20 that harbors BCAS1 gene. FISH assay against the BCAS1 gene (red) and the centromere of chromosome 20 (green). (**A**) Representative FISH image showing the presence of four copies of chromosome 20 (green) in OG. (**B**) Representative FISH image showing the presence of three copies of chromosome 20 (green) in GB. (**C**) Representative FISH image showing the presence of three copies of chromosome 20 (green) in AS. *n* = 1 OG, *n* = 1 AS, *n* = 1 GB. Scale bars: 5 μm.

Genetic alterations, including chromosomal abnormalities, have been described as enhancers of tumor malignancy in diffuse gliomas [28, 29]. Given that *BCAS1* gene is located in chromosome 20, we performed FISH using a reference centromeric probe of chromosome 20 in OG, GB and AS samples (Figure 7). Our results revealed frequent alterations in the number of chromosomes 20 associated with aneuploidy condition in all analyzed samples. However, we did not observed amplification of *BCAS1* gene. Further studies are needed to identify changes in the DNA sequence of BCAS1 in diffuse glioma.

## DISCUSSION

Diffuse gliomas are the most common tumors of the adult CNS with a high rate of morbidity and mortality. Current treatments are invasive (surgery, radiotherapy and chemotherapy) and ineffective for long term survival of high-grade tumor patients [2, 4, 7]. Latest research efforts focus on identifying new biomarkers and targets to advance the knowledge on the molecular aspects to be considered in the classification of gliomas. This line of research will improve the diagnosis timing and the development of new treatments [30]. Here we show the first study evaluating the expression profile of BCAS1 protein in a large cohort of diffuse glioma patients (17 OG, 60 GB and 8 AS). In addition, we provide a fine characterization of the morphological and ultrastructural features of BCAS1-expressing cells and their association with proliferation status.

Our results uncover that primary diffuse gliomas contain BCAS1^+^ cells that can exhibit two different morphologies: stellate and spherical. Interestingly, stellate and spherical BCAS1^+^ cells within the tumors differ from the BCAS1-expressing oligodendrocytes that were previously described in the white matter tissue [17]. Immature oligodendrocytes in the white matter display scant cytoplasm surrounding a rounded nucleus and highly branched processes that *contact de novo* synthesized myelin. In contrast, tumor BCAS1-expressing cells do not show a specific localization, do not contact myelin and their location can be dispersed throughout the tumor, forming small groups, or forming large clusters in the case of grade 3 OG. These results indicate that tumor BCAS1^+^ cells are not myelinating oligodendrocytes. However, we found that tumor BCAS1^+^ cells express other oligodendroglial markers (i.e., OLIG2 and SOX10) related to immature stages within the oligodendrocyte linage. Previous studies have linked the expression of oligodendrocyte progenitors (OPC-like) markers to tumor aggressiveness in OG and GB [8, 31–33]. This, together with the co-expression of EGFR marker, associated with high malignancy gliomas [34], supports the possible tumorigenic capacity that the BCAS1^+^ population could display.

The fact that we identified two morphologically different cell types with unique ultrastructural characteristics expressing BCAS1 raises the question of whether stellate and spherical cells are the same population in different activation states, or they are two different populations within the tumor. Previous studies show that cells presenting proliferative capacity and large cytoplasm are related to activated states [35], while non-proliferative cells with scant cytoplasm correspond to a dormant or quiescent state in the tumor cells [36, 37]. In addition, there are differences on the chromatin compaction which lead a quiescent cell to an abnormal cell state [38]. Concretely, chromatin arrangement and the decrease of nucleus stiffness is a common tumor cell trait [39]. According to our results, we suggest stellate BCAS1^+^ cells display similar morphological and ultrastructural characteristics corresponding to an activated cell state, because their large size, abundant cytoplasm, present numerous prolongations. In addition, unlike oligodendrocytes, which have a classical condensed chromatin contacting the inner nuclear membrane [17], spherical BCAS1^+^ cells in the tumor exhibit loose chromatin that does not contact the nuclear membrane. In contrast, spherical BCAS1^+^ cells have a quiescent state, with a smaller size, scarce cytoplasm without prolongations and a higher stiffness in the nuclei.

Despite AS is a type of diffuse glioma, the cellular ultrastructural and molecular characteristics of BCAS1^+^ cells differ from that found in OG and GB. Interestingly, the BCAS1-expressing cells show a completely different ultrastructure, which resembles to their non-neoplastic astrocytic counterpart given the abundance of intermediate filaments in their cytoplasm, where the expression of BCAS1 was found. Moreover, BCAS1^+^ cells in AS do not proliferate and not only co-express OLIG2 and SOX10, but also GFAP, an astrocyte-related intermediate filament marker characteristic of their non-neoplastic counterparts, astrocytes [22]. This reveals that, in gliomas, neoplastic cells could re-express immature markers, such as astrocytes do in inflammatory conditions, as a dedifferentiation mechanism [40]. In other non-CNS tumors, the re-expression of stem cell markers has been detected as a pro-tumorigenic feature [41].

In diffuse gliomas, the intratumoral heterogeneity is one of the challenges to develop therapeutic strategies [42]. Thus, identifying proliferative populations is of great interest for the field. In this study, we have identified a BCAS1^+^ cell population with proliferative capacity, suggesting its potential to be used as a biomarker for tumor malignancy. The capacity of the BCAS1^+^ cells to form nodules and its location close to the blood vessels in GB evokes a mechanism to glioma progression [43]. In line with other studies [8, 44], our results suggest that OPC markers can be linked to aggressiveness or aggressive populations in diffuse gliomas. The fact of having detected more grade 3 OG samples with BCAS1^+^ nodules, increase the interest of further elucidate the function of BCAS1^+^ cell population in those tumors. BCAS1 upregulation has been recently associated with migration, proliferation and recurrence in GB [16, 45]. Finally, although the expression of BCAS1 has been associated with prognosis in different cancers, including prostate [11], pancreatic [12], colorectal [13] and gastric cancer [14], more studies are needed to fully understand how BCAS1 expression affects the function of neoplastic cells in diffuse gliomas, including its role in tumorigenesis. In addition, the reduced number of samples that we used for some procedures (e.g. immunostaining quantification, FISH) is a limitation of our study that has to be taken into consideration.

In summary, this is the first study describing a BCAS1^+^ cell population in a large cohort of diffuse glioma patients. Despite the two morphologically and functionally different BCAS1^+^ cells found, the most interesting fact was the capacity of BCAS1^+^ cells to form compact nodules and the proliferative capacity of the stellate BCAS1^+^ cells. These cellular traits lead us to question if BCAS1-expressing cells could play a role in tumor aggressiveness, particularly in OG. Future studies modifying the expression of BCAS1 *in vitro* are necessary to determine if this protein can act as a regulator of tumorigenesis. A thorough comprehension of the role of BCAS1 not only in diffuse gliomas but also in other type of brain tumors would help to unravel its significance in tumor initiation, progression, and invasion. In conclusion, this insight will shed light on the establishment of BCAS1 as a clinically relevant molecule, serving not only as a diagnostic or prognostic marker but also as a novel therapeutic target for the development of cutting-edge treatments.

## MATERIALS AND METHODS

### Human brain tissue collection

We analyzed a total of 85 primary diffuse glioma biopsies, collected from 85 different patients (17 OG, 60 GB and 8 AS), obtained through surgical resection performed at the Hospital Universitari i Politècnic la Fe, between 2015 and 2022. To compare the cellular traits between BCAS1-expressing neoplastic cells and non-neoplastic immature oligodendrocytes, we included 3 non-tumor control samples (i.e., 3 samples of non-affected white matter from resections of focal cortical dysplasia in 3 different patients). Samples were collected following appropriate informed consent and in strict accordance with the ethical standards of the committee of responsibility for experimentation in humans (institutional and national) and considering the Declaration of Helsinki of 1964 and its subsequent modifications. All surgically removed tissues were obtained via *en bloc* extraction or surgical suction from the core of the tumor. Tissue was fixed in 4% paraformaldehyde in 0.1 M phosphate buffer, pH 7.4 (PB) for 24 h and subsequently embedded in paraffin. Sections were prepared to define representative tumor regions, and tumor diagnosis was confirmed through histopathological and molecular analyzes according to the WHO classification [2, 5] (see Supplementary Figure 4). Furthermore, we analyze ATRX expression, TP53 and TERT promoters’ mutation, and MGMT promoter methylation (see Supplementary Table 3). Only biopsies obtained at the primary diagnosis were considered, ensuring that patients were not undergoing any treatment at the time of sample collection. Information about the samples included in this study is provided in the Supplementary Table 3.

### Histology

OG and AS paraffin-embedded samples were cut in 5 μm sections using a Microm HM340E microtome (Thermo Fisher Scientific, Waltham, MA, USA) and slides were stored at room temperature until immunostaining was performed. GB samples were organized in tissue microarrays, with at least three to four cores from each tumor, representing morphological diverse zones. Standard Hematoxylin and Eosin staining was also performed for pathological evaluation together with IDH-status and ATRX analysis.

### Immunohistochemistry and immunofluorescence

Tumor sections were deparaffinized in xylene and rehydrated in decreasing concentrations of ethanol. For heat-induced antigen retrieval, samples were transferred to a 1:200 Immunosaver solution (Electron Microscopy Sciences, Hatfield, PA, USA) in doble distillated water using a 2100 Retriever (Prestige Medical, Lancashire, United Kingdom). To block endogenous peroxidase activity, samples were incubated in a 10% methanol and 0.3% hydrogen peroxide solution for 15 min. Then, they were blocked and permeabilized during 1 h at room temperature using 5% normal goat serum (Gibco, USA), 0.1% Triton X-100 and 0.1% albumin in 0.1 M PB. Subsequently, samples were incubated in primary antibodies overnight at 4°C (Table 1). For immunohistochemistry, samples were incubated with biotinylated secondary antibody for 1 h. For signal amplification, slides were washed and then incubated with the Vectastain Elite ABC kit (Vector) during 1 h at room temperature. The slides were revealed with 3,3′-Diaminobenzidine (DAB, Merck, Darmstadt, Germany) and then counterstained in a 1% cresyl violet solution. Finally, samples were dehydrated in increasing grades of ethanol, washed with xylene, and mounted using Eukitt medium (Electron Microscopy Sciences). A Nikon Eclipse E800 microscope was used for imaging.

**Table 1:** Antibodies used for the different immunohistochemical techniques performed

		Primary antibody					Secondary antibody				
		Marker	Company	Reference	Animal	Dilution	Marker	Company	Reference	Animal	Dilution
IMMUNOHISTO-CHEMISTRY		NABC1 (BCAS1)	Santa Cruz Biotechnology (Dallas, TX, USA)	sc-136342	mouse	1:200	biotinylated IgG	Vector (Burlingame, CA, USA)	BA 9200	Goat anti-mouse	1:400
IMMUNOFLUORESCENCE	BCAS1/EGFR/VIM	NABC1 (BCAS1)	Santa Cruz Biotechnology (Dallas, TX, USA)	sc-136342	mouse	1:200	Alexa 647	Invitrogen (Carlsbad, CA, USA)	A31571	Donkey anti-mouse	1:400
		EGFR	Millipore (Burlington, MA, USA)	06-847	rabbit	1:200	Alexa 555	Invitrogen (Carlsbad, CA, USA)	A21428	Goat anti-rabbit	1:400
		VIM	Abcam (Cambridge, United Kingdom)	ab73159	chicken	1:300	Alexa 488	Invitrogen (Carlsbad, CA, USA)	A11039	Goat anti-chicken	1:400
	BCAS1/KI67 BCAS1/OLIG2 BCAS1/SOX10 BCAS1/CXCR4 BCAS1/BLBP BCAS1/GFAP	NABC1 (BCAS1)	Santa Cruz Biotechnology (Dallas, TX, USA)	sc-136342	mouse	1:200	Alexa 488	Invitrogen (Carlsbad, CA, USA)	A11001	Goat anti-mouse	1:400
		KI67	Abcam (Cambridge, United Kingdom)	ab16667	rabbit	1:150	Alexa 555	Invitrogen (Carlsbad, CA, USA)	A21428	Goat anti-rabbit	1:400
		OLIG2	Millipore (Burlington, MA, USA)	AB9610	rabbit	1:250					
		SOX10	Abcam (Cambridge, United Kingdom)	ab155271	rabbit	1:300					
		CXCR4	Abcam (Cambridge, United Kingdom)	ab124824	rabbit	1:100					
		BLBP	Millipore (Burlington, MA, USA)	ABN14	rabbit	1:150					
		GFAP	Dako (Glostrup, Denmark)	Z0334	rabbit	1:500					
IMMUNOGOLD		NABC1 (BCAS1)	Santa Cruz Biotechnology (Dallas, TX, USA)	sc-136342	mouse	1:200	biotinylated IgG	Vector (Burlingame, CA, USA)	BA 9200	Goat anti-mouse	1:300
		Biotin	Jackson Immuno Research (Cambridge, United Kingdom)	200-002-211	mouse	1:100	colloidal gold-conjugated antibody	Electron Microscopy Sciences (Hatfield, PA, USA)	25212	Goat anti-mouse	1:50

The Table 1 shows, for each immunohistochemical technique performed, the information of the different primary antibodies used (five columns on the left, “Primary antibody”) and the information of the corresponding secondary antibody (five columns on the right, “Secondary antibody”).

For immunofluorescence, samples were incubated in fluorochrome-conjugated secondary antibodies (Table 1). After 1 h of incubation at room temperature, sections were washed and counterstained with DAPI (1:1000, Invitrogen, Carlsbad, CA, USA) for 10 min. Finally, slides were mounted using FluorSave^™^ medium (Millipore, Burlington, MA, USA) and analyzed using a Nikon i80 microscope and a Nikon CCD DS-Qi1Mc digital camera.

### Immunogold labelling

Tyramide signal amplification-immunogold labelling was carried out as previously described [46]. Diffuse glioma tissues were cut in 50 μm sections using a Leica VT1000S vibratome (Leica Biosystems, Wetzlar, Germany). Slices were treated with 1% sodium borohydride. Then, tissue was permeabilized by freeze–thaw cycles in 2-methylbutane at −60°C. Samples were incubated in the primary antibody solution against BCAS1 for 72 h at 4°C (see Table 1 for antibody information). Secondary biotinylated antibody incubation was performed for 2 h 30 min at room temperature. Then, sections were incubated in Streptavidin-HRP (SA-5004, Vector) and in biotin-tyramide solution (SAT700001EA, Perkin Elmer, Shelton, CT, USA). After washing, samples were blocked in 0.5% acetylated bovine serum albumin (Aurion, Wageningen, The Netherlands) and incubated in anti-biotin antibody overnight at 4ºC. The next day, samples were incubated with a colloidal gold-conjugated secondary antibody for 2 h at room temperature. Silver enhancement was performed using the R-Gent SE-LM kit (500.011, Aurion). Subsequently, samples were immersed in gold toning solution (HT-1004, Sigma-Aldrich, USA) for 10 min. To stabilize the gold-silver bonds, sections were immersed on 0.3% sodium thiosulfate solution, and then they were post-fixed in a solution of 2% glutaraldehyde. Finally, repetitive washes with 0.1 M PB were performed before epoxy resin embedding.

### Tissue processing for transmission electron microscopy analysis

Samples were post-fixed with a solution of 1% osmium tetroxide and 7% glucose in 0.1 M PB for 30 min at room temperature, washed with deionized water and partially dehydrated in increasing concentrations of ethanol. Then, sections were contrasted with 2% uranyl acetate in 70% ethanol for 2 h 30 min at 4°C. Subsequently, dehydration with 70%, 96%, 100% ethanol, and propylene oxide was carried out. Sections were embedded in Durcupan epoxy resin (Sigma-Aldrich), where they remained overnight at room temperature in gentle shaking. The next day, samples were transferred to flat molds with fresh resin followed by 72 h at 70ºC to resin polymerization. Once the resin had polymerized, the region of interest (zone with BCAS1^+^ cells) in each section was selected and semithin sections (1.5 μm) were obtained using a UC7 ultramicrotome (Leica). After staining the sections with 1% toluidine blue solution, ultrathin sections (70–80 nm) were obtained. Finally, images were taken at 80 kV on a FEI Tecnai G^2^ Spirit transmission electron microscope (FEI Company, Hillsboro, OR) equipped with a Xarosa (20 Megapixel resolution) digital camera using Radius image acquisition software (EMSIS GmbH, Münster, Germany).

### Fluorescence *in situ* hybridization

Fluorescence *in situ* hybridization (FISH) assay with a BCAS1 probe (RP11-705A3, red labeling) and a Chromosome 20 Centromere probe (CHR20-10-RE, green labeling) (Empire Genomics, Buffalo, NY, USA) was performed in a sample of each tumor type studied. After deparaffinizing and rehydrating 2.5 μm tissue section, manufacturers’ automated hybridization protocol by Empire Genomics was carried out using the HYBrite platform (Abbott Molecular, Des Plaines, IL, USA). Cell nuclei were counterstained with DAPI. Fluorescence was analyzed using an Axioscope 5 microscope equipped with Axiocam 305 mono camera (ZEISS). For each sample, more than 100 nuclei were counted. Aneuploidy was defined as a chromosome count that differed from the normal 2 n chromosome number.

### Stereological quantification

To estimate cell density and proliferative rate for BCAS1^+^ cells, stereological quantification was performed. In each tumor analyzed, 10 different 40× fields from ‘hot spots’ were studied. ‘Hot spots’ were defined as tumor regions in which more BCAS1^+^ cells were detected. In the case of GB, the tumors with less than 3 representative areas were excluded from the study. We used ‘Multi-point’ tool ImageJ for quantification. Double stained cells in proliferation studies (BCAS1^+^, KI67^+^) were counted manually.

### Statistical analysis

Statistical analyses were performed using GraphPad Prism 5.0 software (GraphPad Software Inc, San Diego, CA, USA). Kolmogorov-Smirnov test was applied to check the normality of all data sets. As all values analyzed shown a non-normal distribution, the different data sets were analyzed with non-parametric Mann–Whitney test to compare two independent groups or with the non-parametric Kruskal–Wallis test followed by Dunn’s correction for multiple comparisons, to compare more than two independent groups. Center lines in the boxes indicate the median and whiskers indicate the minimum and the maximum values. *p* value < 0.05 was established for statistical significance.

## SUPPLEMENTARY MATERIALS



## Abbreviations

ATRX: Alpha thalassemia/mental retardation syndrome X-linked
AS: Astrocytoma
BCAS1/NABC1: Breast carcinoma amplified sequence 1
BLBP: Brain lipid binding protein
CNS: Central nervous system
CXCR4: C-X-C chemokine receptor type 4
DAB: 3,3′-Diaminobenzidine
EGFR: Epidermal growth factor receptor
FISH: Fluorescence *in situ* hybridization
GB: Glioblastoma
GFAP: *Glial Fibrillary Acidic Protein*
IDH: Isocitrate dehydrogenase
OG: Oligodendroglioma
OLIG2: Oligodendrocyte transcription factor 2
OPC: Oligodendrocyte precursor cell
PB: Phosphate buffer
RER: Rough endoplasmic reticulum
SOX10: SRY-Box Transcription Factor 10
VIM: Vimentin
WHO: World Health Organization
