# Supplementary material for: BCAS1 defines a heterogeneous cell population in diffuse gliomas
Source: Oncotarget. 2024 Jan 24;15:49–64. doi: 10.18632/oncotarget.28553 (PMC10812236; doi:10.18632/oncotarget.28553)
Supplement: Supplementary file 1 [file oncotarget-15-28553-s001.pdf]

BCAS1 defines a heterogeneous cell population in diffuse gliomas

SUPPLEMENTARY MATERIALS

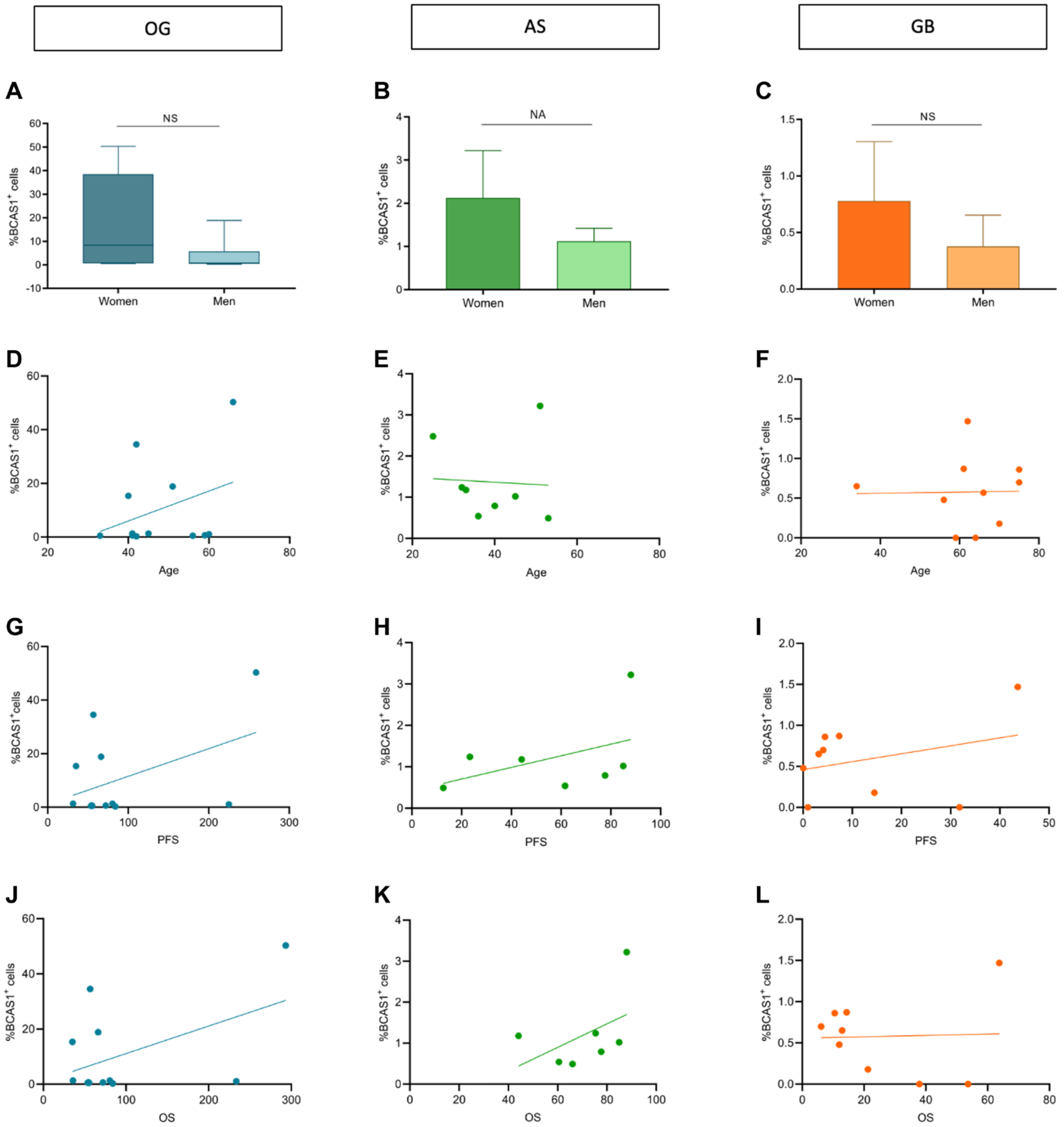

Supplementary Figure 1: The percentage of BCAS1$^+$ cells does not correlate with the sex or the age of patient with diffuse glioma. Comparisons between the percentage of BCASl$^+$ cells and the sex (**A**–**C**), the age (**D**–**F**), the progression-free survival (PFS) (**G**–**I**) or the overall survival (OS) (**J**–**L**) of the patients. (A) Percentage of BCASl$^+$ cells in women and men with OG. $n = 6$ women, $n = 6$ men; Mann- Whitney test; p-value = 0.2403. (B) Percentage of BCASl$^+$ cells in women and men with AS. $n = 2$ women, $n = 6$ men. (C) Percentage of BCASl$^+$ cells in women and men with GB. $n = 5$ women, $n = 5$ men; Unpaired t test; p-value = 0.1679. (D) Percentage of BCAS1$^+$ cells front the age of patients with OG. Trend line $R^2 = 0.1166$; p-value = 0.2774. (E) Percentage of BCASl$^+$ cells front the age of patients with AS. Trend line $R^2 = 0.0032$; p-value = 0.8930. (F) Percentage of BCASl$^+$ cells front the age of patients with GB. Trend line $R^2 = 0.0004$; p-value = 0.9590. (G) Percentage of BCASl$^+$ cells front the PFS of patients with OG. Trend line $R^2 = 0.2093$; p-value = 0.1348. (H) Percentage of BCAS1$^+$ cells front the PFS of patients with AS. Trend line $R^2 = 0.2059$; p-value = 0.3065. (I) Percentage of BCASl$^+$ cells front the PFS of patients with GB. Trend line $R^2 = 0.09723$; p-value = 0.4140. (J) Percentage of BCASl$^+$ cells front the OS of patients with OG. Trend line $R^2 = 0.2439$; p-value = 0.1027. (K) Percentage of BCAS1$^+$ cells front the OS of patients with AS. Trend line $R^2 = 0.2211$; p-value = 0.2869. (L) Percentage of BCASl$^+$ cells front the OS of patients with GB. Trend line $R^2 = 0.001325$; p-value = 0.9259. Center lines in the boxes indicate the median and whiskers indicate the minimum and the maximum values. Abbreviation: NS: Not significant. NA: Statistical analysis was not performed because $n = 2$.

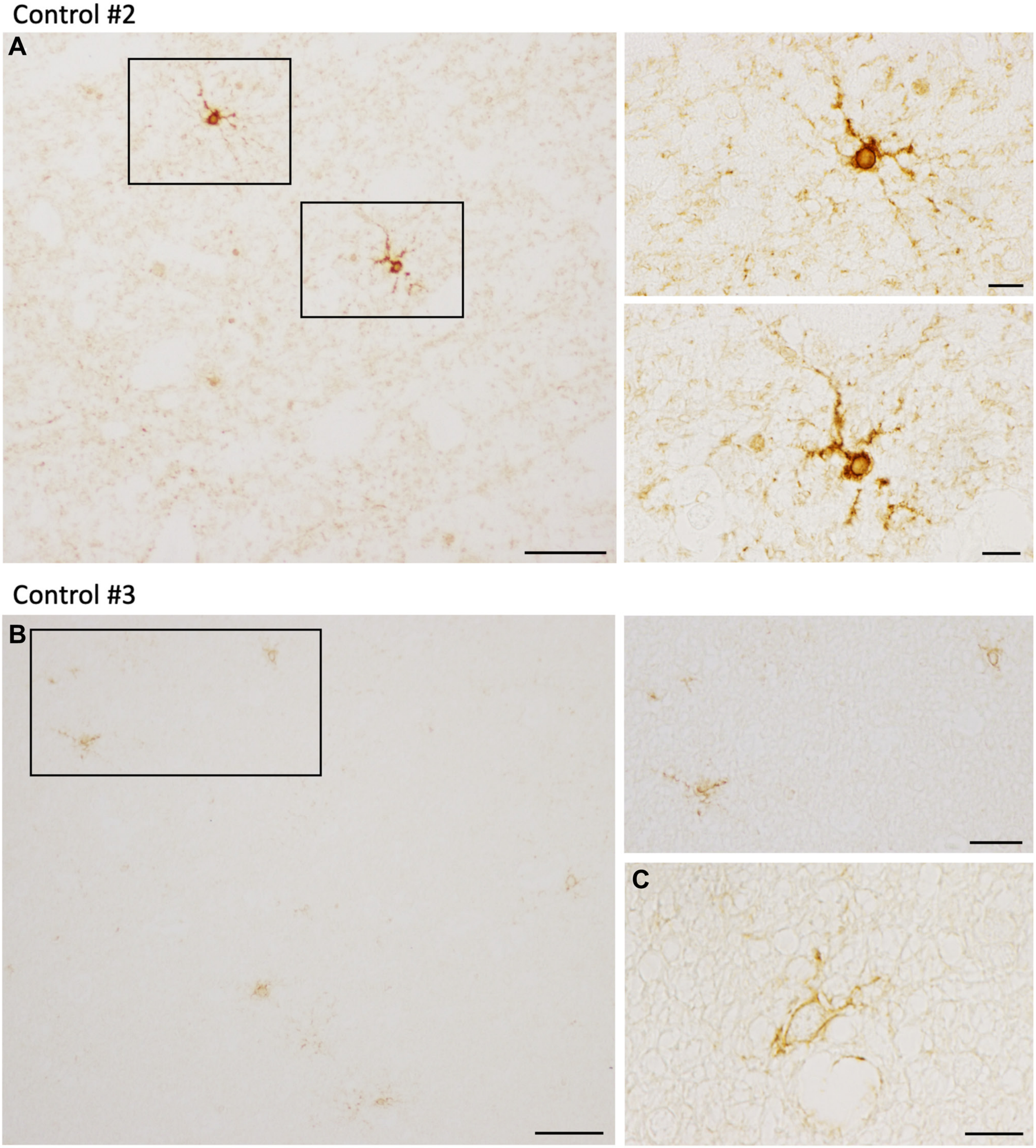

Supplementary Figure 2: BCAS1^{+} cells in non-tumoral human brain tissues. Immunohistochemistry against BCASl in two additional control samples (non-tumoral human brain tissue). All BCASl^{+} cells found in control samples present stellate morphology with a small nucleus and numerous branched cytoplasmic expansions. (**A**) BCAS1^{+} cells from a control brain human tissue (control #2). Inserts show high magnification of BCAS1^{+} cells. (**B**) BCAS 1^{+} cells from another control human brain tissue (control #3). The insert shows high magnification of BCASl^{+} cells. (**C**) Detail of a BCASl^{+} cell with stellate morphology in the control #3. Scale bars: A panoramic, 50 µm; A insert, 10 µm; B panoramic, 50 µm; B insert, 25 µm; C, 10 µm.

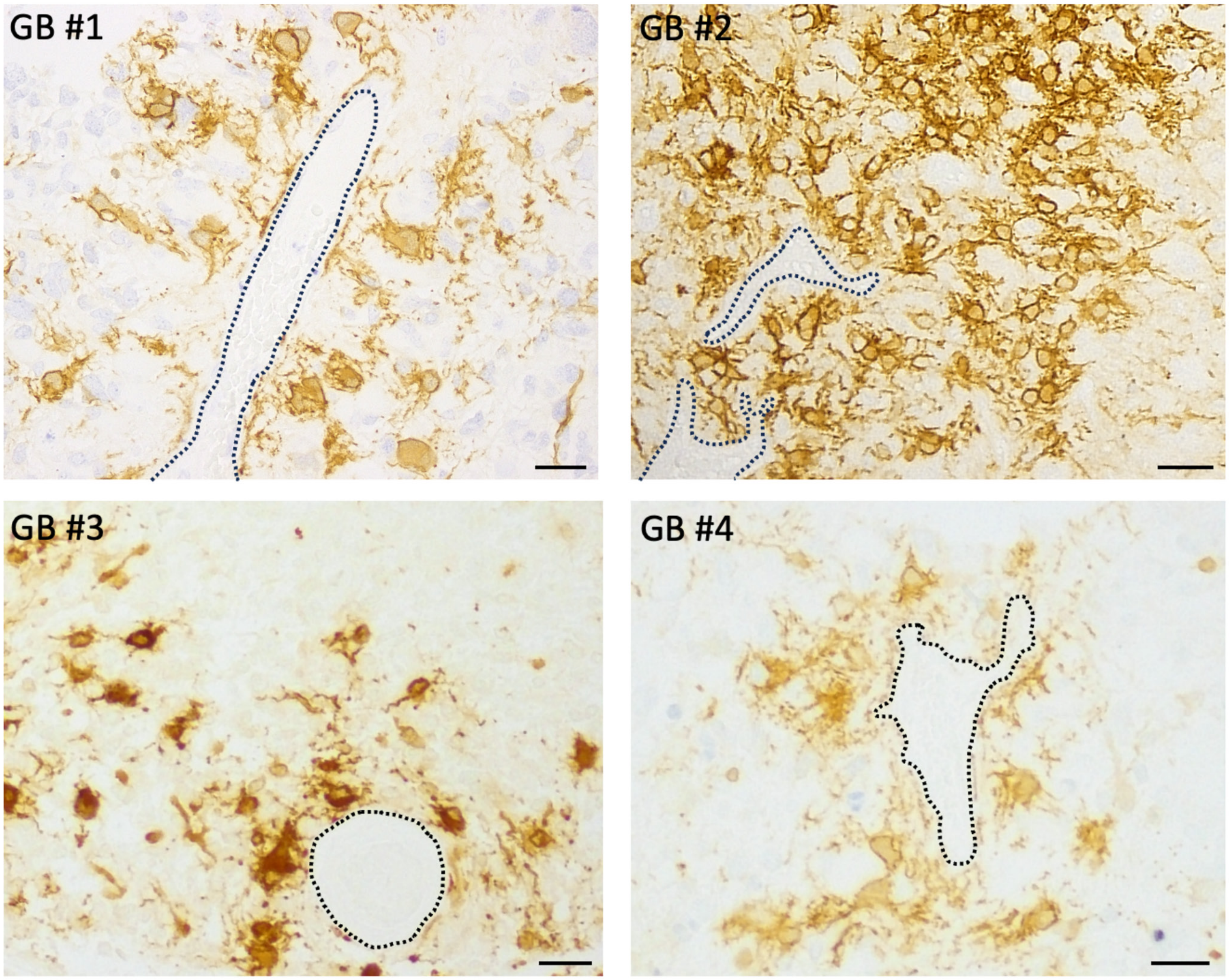

Supplementary Figure 3: BCASl⁺ cells are located in close proximity to the blood vessels in GB: DAB immunohistochemistry against BCASl in GB samples from 4 different patients. Note that BCASl⁺ cells (brown) are located in close vicinity to blood vessels. All blood vessels have been delineated by a dotted line. Scale bars: 25 µm.

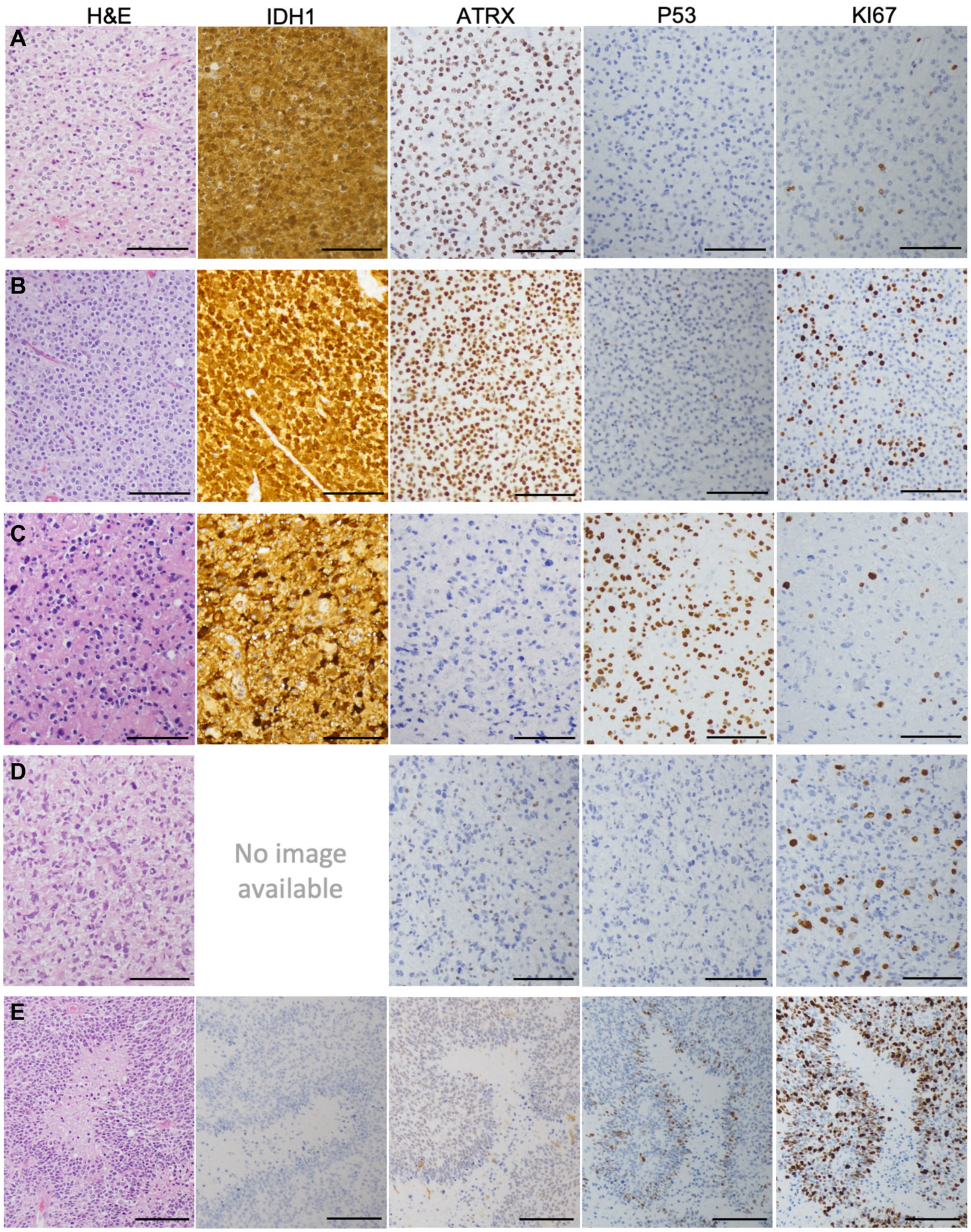

Supplementary Figure 4: Representative images of the immunohistochemical staining obtained for diagnosing of the tumors included in the study: Individual immunostainings are indicated on top of each figure. DAB (brown) was used for visualization of antibody binding. Nuclei were counterstained with cresyl violet (blue). (**A**) Grade 2 oligodendroglioma; IDHl mutant, but ATRX and P53 wild type. Hematoxylin and eosin (H&E) shows the classic "fried egg" appearance. IDHl FISH assay detected lp/19q codeletion in all grade 2 oligodendroglioma studied. (**B**) Grade 3 oligodendroglioma: same as oligodendroglioma grade 2, but higher cellular density and proliferation (K167). IDHl FISH assay detected lp/19q codeletion in all grade 3 oligodendroglioma studied. (**C**) Grade 2 astrocytoma: IDHl mutant, loss of nuclear ATRX expression and strong nuclear immunoreactivity with antibody against P53. (**D**) Grade 3 astrocytoma: same as astrocytoma grade 2, but higher cellular density and proliferation. (**E**) Glioblastoma: lack of IDHl positivity, loss of nuclear ATRX expression and a lot of proliferation. Typical structures of pseudopalisading necrosis are observed in all glioblastoma analyzed. Scale bars: 100 μm.

Supplementary Table 1: Results of the different quantifications performed

Tumor ID	%BCAS1^{+} TOTAL	% Stellate BCAS1^{+} cells	% Spherical BCAS1^{+} cells	% (BCAS1^{+}/Ki67^{+})/ BCAS1^{+} cells	% (BCAS1^{+}/Ki67^{+})/ KI67^{+} cells	% (BCAS1^{+}/Ki67^{+})/ DAPI^{+} cells
**OG 1**	50.31	100	0	22.84	65.77	11.49
**OG 2**	0.27	23.81	76.19	0	0	0
**OG 3**	1.33	0	100	0	0	0
**OG 4**	0.60	38.10	61.90	0	0	0
**OG 5**	18.85	100	0	1.22	12.24	0.23
**OG 6**	0.51	0	100	0	0	0
**OG 7**	34.51	100	0	0.59	28.57	0.20
**OG 8**	0.53	50	50	8.33	0.93	0.02
**OG 9**	1.32	7.32	92.68	33.33	0.60	0.03
**OG 10**	0.69	73.68	26.32	0	0	0
**OG 11**	1.05	0	100	0	0	0
**OG 12**	15.33	100	0	7.64	18.10	1.17
**AS 1**	1.02	17.86	82.14	0	0	0
**AS 2**	3.22	24.04	75.96	0	0	0
**AS 3**	0.49	0	100	0	0	0
**AS 4**	2.48	94.52	5.48	0	0	0
**AS 5**	0.79	50	50	0	0	0
**AS 6**	1.24	0	100	0	0	0
**AS 7**	1.18	37.04	62.96	0	0	0
**AS 8**	0.54	43.48	56.52	0	0	0
**GB 1**	0.65	10.71	89.29	0	0	0
**GB 2**	1.47	0	100	0	0	0
**GB 3**	0	0	0	0	0	0
**GB 4**	0.86	0	100	0	0	0
**GB 5**	0.57	0	100	0	0	0
**GB 6**	0.48	0	100	0	0	0
**GB 7**	0.87	1.69	98.31	0	0	0
**GB 8**	0	0	0	0	0	0
**GB 9**	0.70	3.45	96.55	0	0	0
**GB 10**	0.18	0	100	0	0	0

Values of all quantification performed to estimate the total percentage of BCAS1^{+} cells in each tumor analyzed (%BCAS1^{+} TOTAL), the percentage of stellate or spherical BCAS1^{+} cells (%Stellate BCAS1^{+} cells and %Spherical BCAS1^{+} cells, respectively), and the number of proliferating stellate BCAS1-expressing cells with respect to the total BCAS1^{+} cells (%(BCAS1^{+}/Ki67^{+})/BCAS1^{+} cells), total proliferating cells (%(BCAS1^{+}/Ki67^{+})/KI67^{+} cells) or total cells in the analyzed region (%(BCAS1^{+}/Ki67^{+})/DAPI^{+} cells).

Supplementary Table 2: Antigens studied and their characteristics

Antigen	Antigen characteristics	References	Purpose
VIM	Marker of motility and invasiveness in cancer	[26]	Microenvironment and cellular traits
GFAP	Marker of astrocyte lineage	[22]	Microenvironment and cellular traits
EGFR	Marker of cell migration, adhesion and proliferation	[25]	Microenvironment and maturation state
OLIG2	Marker of oligodendrocyte lineage	[21]	Maturation state
SOX10	Transcription factor expressed in oligodendrocyte and other glial precursors	[19]	Maturation state
CXCR4	Marker of cell proliferation in gliomas	[20]	Cellular traits
BLBP	Marker of cell proliferation in gliomas	[18]	Cellular traits

Information on selected antigens to study in this research and the reference in the bibliography where more information on this molecule could be found.

Supplementary Table 3: Clinical data of patients and histopathological data of tumors

Case ID	Sex	Age at surgery (years)	Histopathological diagnosis	WHO Grade	Localization	Molecular profile						Progression-free survival (months)	Overall survival (months)
						1p/19q	IDH1	ATRX	TP53	MGMT	TERT		
1	F	66	Anaplastic Oligodendroglioma	3	Right parietal-occipital	Codeletion	Mut	NMut	NMut	Met	Mut	258.85	293.32
2	M	42	Anaplastic Oligodendroglioma	3	Right temporal	Codeletion	Mut	NMut	NMut	Met	Mut	83.93	83.93
3	M	41	Diffuse Oligodendroglioma	2	Left frontal	Codeletion	Mut	NMut	NMut	Met	Mut	80.38	80.38
4	M	41	Anaplastic Oligodendroglioma	3	Right parietal	Codeletion	Mut	ND	NMut	Met	Mut	72.13	72.13
5	M	51	Anaplastic Oligodendroglioma	3	Right parietal	Codeletion	Mut	NMut	NMut	Met	Mut	66.24	66.24
6	M	56	Diffuse Oligodendroglioma	2	Left frontal	Codeletion	Mut	NMut	NMut	Met	Mut	55.35	55.35
7	F	42	Diffuse Oligodendroglioma	2	Left parietal	Codeletion	Mut	NMut	NMut	Met	Mut	56.60	56.60
8	M	31	Anaplastic Oligodendroglioma	3	Right insula	Codeletion	Mut	NMut	NMut	Met	Mut	120.13	178.61
9	F	33	Diffuse Oligodendroglioma	2	Left parietal	Codeletion	Mut	NMut	NMut	Met	Mut	53.84	53.84
10	F	45	Anaplastic Oligodendroglioma	3	Left frontal	Codeletion	Mut	NMut	NMut	Met	Mut	31.51	35.94
11	F	59	Diffuse Oligodendroglioma	2	Right frontal	Codeletion	Mut	NMut	NMut	Met	Mut	54.43	54.43
12	M	60	Diffuse Oligodendroglioma	2	Left frontal	Codeletion	Mut	NMut	NMut	Met	Mut	225.00	233.57
13	F	28	Anaplastic Oligodendroglioma	3	Right frontal-parietal	Codeletion	Mut	NMut	NMut	Met	Mut	35.91	35.91
14	F	57	Anaplastic Oligodendroglioma	3	Left temporal	Codeletion	Mut	NMut	NMut	Met	Mut	47.06	47.06
15	F	40	Anaplastic Oligodendroglioma	3	Left frontal	Codeletion	Mut	NMut	NMut	Met	Mut	35.12	35.12
16	M	49	Diffuse Oligodendroglioma	2	Right frontal	Codeletion	Mut	NMut	NMut	NMet	Mut	32.88	32.88
17	F	45	Diffuse Oligodendroglioma	2	Right frontal	Codeletion	Mut	NMut	NMut	Met	Mut	53.97	53.97
18	M	53	Glioblastoma	4	Left temporal	Not deleted	NMut	NMut	Mut	NMet	Mut	4.01	8.03

Case ID	Sex	Age at surgery (years)	Histopathological diagnosis	WHO Grade	Localization	Molecular profile						Progression-free survival (months)	Overall survival (months)
						1p/19q	IDH1	ATRX	TP53	MGMT	TERT		
19	M	34	Glioblastoma	4	Right frontal	Not deleted	NMut	NMut	Mut	NMet	NMut	3.19	12.93
20	F	62	Glioblastoma	4	Left parietal	Not deleted	NMut	NMut	NMut	NMet	NMut	43.65	63.75
21	F	67	Glioblastoma	4	Right temporal	Not deleted	NMut	NMut	NMut	NMet	Mut	NA	16.15
22	M	59	Glioblastoma	4	Right parietal	Not deleted	NMut	NMut	Mut	Met	Mut	31.81	53.68
23	F	75	Glioblastoma	4	Right temporal	Not deleted	NMut	NMut	NMut	Met	Mut	4.47	10.49
24	M	66	Glioblastoma	4	Right frontal	Not deleted	NMut	NMut	NMut	Met	NMut	NA	ND
25	M	56	Glioblastoma	4	Right frontal	Not deleted	NMut	NMut	NMut	NMet	Mut	0.03	11.97
26	M	64	Glioblastoma	4	Right frontal	Not deleted	NMut	NMut	NMut	NMet	Mut	8.55	11.71
27	M	60	Glioblastoma	4	Right temporal	Not deleted	NMut	NMut	NMut	NMet	Mut	31.48	47.01
28	F	61	Glioblastoma	4	Left temporal	Not deleted	NMut	NMut	NMut	NMet	Mut	7.34	14.38
29	F	64	Glioblastoma	4	Right frontal	Not deleted	NMut	NMut	Mut	Met	Mut	0.99	37.93
30	F	75	Glioblastoma	4	Right frontal	Not deleted	NMut	NMut	NMut	NMet	Mut	4.11	6.12
31	F	66	Glioblastoma	4	Left frontal	Not deleted	NMut	NMut	NMut	NMet	Mut	36.28	49.34
32	F	76	Glioblastoma	4	Right temporal	Not deleted	NMut	NMut	Mut	NMet	Mut	0.99	2.07
33	M	70	Glioblastoma	4	Right frontal	Not deleted	NMut	NMut	NMut	Met	Mut	14.51	21.28
34	F	69	Glioblastoma	4	Right parietal	Not deleted	NMut	NMut	Mut	NMet	Mut	3.06	3.65
35	F	71	Glioblastoma	4	Right temporal	Not deleted	NMut	NMut	NMut	NMet	Mut	NA	1.78
36	F	48	Glioblastoma	4	Right frontal	Not deleted	NMut	NMut	NMut	NMet	Mut	3.29	13.19

Case ID	Sex	Age at surgery (years)	Histopathological diagnosis	WHO Grade	Localization	Molecular profile						Progression-free survival (months)	Overall survival (months)
						1p/19q	IDH1	ATRX	TP53	MGMT	TERT		
37	M	71	Glioblastoma	4	Right frontal	Not deleted	NMut	NMut	NMut	NMet	Mut	8.78	8.98
38	M	74	Glioblastoma	4	Right occipital	Not deleted	NMut	NMut	NMut	NMet	Mut	2.01	4.01
39	M	77	Glioblastoma	4	Left frontal	Not deleted	NMut	NMut	NMut	NMet	Mut	15.53	17.50
40	M	63	Glioblastoma	4	Left parietal	Not deleted	NMut	NMut	NMut	NMet	Mut	9.21	11.81
41	F	53	Glioblastoma	4	Right parietal	Not deleted	NMut	NMut	NMut	NMet	Mut	7.80	12.70
42	M	82	Glioblastoma	4	Left temporal	Not deleted	NMut	NMut	NMut	NMet	Mut	5.30	8.39
43	F	55	Glioblastoma	4	Left parietal	Not deleted	NMut	NMut	NMut	NMet	Mut	6.97	7.96
44	F	70	Glioblastoma	4	Right parietal	Not deleted	NMut	NMut	Mut	NMet	Mut	3.95	13.26
45	F	71	Glioblastoma	4	Right temporal	Not deleted	NMut	NMut	NMut	Met	Mut	3.78	8.19
46	M	68	Glioblastoma	4	Left frontal	Not deleted	NMut	NMut	NMut	NMet	Mut	6.41	7.66
47	F	68	Glioblastoma	4	Left parietal	Not deleted	NMut	NMut	NMut	Met	Mut	9.18	9.18
48	F	68	Glioblastoma	4	Right temporal	Not deleted	NMut	NMut	NMut	Met	Mut	14.87	17.57
49	M	71	Glioblastoma	4	Right temporal	Not deleted	NMut	NMut	NMut	NMet	Mut	9.87	11.28
50	M	56	Glioblastoma	4	Left frontal	Not deleted	NMut	NMut	NMut	NMet	Mut	8.26	12.60
51	M	45	Glioblastoma	4	Right frontal	Not deleted	NMut	NMut	NMut	Met	Mut	7.47	19.87
52	F	75	Glioblastoma	4	Right parietal	Not deleted	NMut	NMut	NMut	NMet	Mut	10.56	12.73
53	F	76	Glioblastoma	4	Left temporal	Not deleted	NMut	NMut	Mut	NMet	Mut	5.86	11.78
54	M	70	Glioblastoma	4	Right parietal	Not deleted	NMut	NMut	Mut	NMet	Mut	3.75	9.11

Case ID	Sex	Age at surgery (years)	Histopathological diagnosis	WHO Grade	Localization	Molecular profile						Progression-free survival (months)	Overall survival (months)
						1p/19q	IDH1	ATRX	TP53	MGMT	TERT		
55	F	65	Glioblastoma	4	Left frontal	Not deleted	NMut	NMut	NMut	NMet	Mut	30.43	59.80
56	M	56	Glioblastoma	4	Right temporal	Not deleted	NMut	NMut	NMut	Met	Mut	7.89	15.99
57	M	73	Glioblastoma	4	Right temporal	Not deleted	NMut	NMut	NMut	NMet	Mut	10.26	11.02
58	M	56	Glioblastoma	4	Right frontal	Not deleted	NMut	NMut	NMut	Met	Mut	31.81	44.64
59	F	45	Glioblastoma	4	Left parietal	Not deleted	NMut	NMut	Mut	Met	Mut	25.63	32.43
60	F	51	Glioblastoma	4	Left temporal	Not deleted	NMut	NMut	NMut	Met	Mut	13.88	15.00
61	F	52	Glioblastoma	4	Right frontal	Not deleted	NMut	NMut	ND	NMet	Mut	4.31	10.69
62	M	55	Glioblastoma	4	Left temporal	Not deleted	NMut	NMut	Mut	NMet	Mut	5.53	10.16
63	F	78	Glioblastoma	4	Left temporal	Not deleted	NMut	NMut	NMut	Met	Mut	2.93	3.22
64	F	65	Glioblastoma	4	Left temporal	Not deleted	NMut	NMut	NMut	Met	Mut	NA	NA
65	F	65	Glioblastoma	4	Right parietal	Not deleted	NMut	NMut	ND	Met	Mut	19.05	24.31
66	M	61	Glioblastoma	4	Left frontal	Not deleted	NMut	NMut	NMut	Met	Mut	12.04	18.26
67	F	38	Glioblastoma	4	Left occipital	Not deleted	NMut	NMut	Mut	NMet	Mut	19.08	33.62
68	M	41	Glioblastoma	4	Right temporal	Not deleted	NMut	NMut	ND	NMet	Mut	4.70	6.88
69	F	52	Glioblastoma	4	Right temporal	Not deleted	NMut	NMut	Mut	NMet	Mut	16.41	18.95
70	F	71	Glioblastoma	4	Right frontal	Not deleted	NMut	NMut	NMut	Met	Mut	8.88	13.55
71	M	71	Glioblastoma	4	Left temporal	Not deleted	NMut	NMut	ND	NMet	Mut	9.51	18.75
72	F	74	Glioblastoma	4	Right frontal	Not deleted	NMut	NMut	NMut	Met	Mut	23.98	24.77

Case ID	Sex	Age at surgery (years)	Histopathological diagnosis	WHO Grade	Localization	Molecular profile						Progression-free survival (months)	Overall survival (months)
						1p/19q	IDH1	ATRX	TP53	MGMT	TERT		
73	F	75	Glioblastoma	4	Right frontal	Not deleted	NMut	NMut	ND	Met	Mut	4.14	6.25
74	M	73	Glioblastoma	4	Right temporal	Not deleted	NMut	NMut	Mut	NMet	Mut	8.91	13.49
75	F	64	Glioblastoma	4	Right occipital	Not deleted	NMut	NMut	ND	NMet	Mut	0.86	3.32
76	M	84	Glioblastoma	4	Right temporal	Not deleted	NMut	NMut	NMut	Met	Mut	3.03	3.03
77	F	73	Glioblastoma	4	Right temporal	Not deleted	NMut	NMut	Mut	NMet	NMut	4.47	6.76
78	F	45	Diffuse Astrocytoma	2	Left frontal	Not deleted	ND	ND	Mut	Met	NMut	84.99	84.99
79	F	51	Diffuse Astrocytoma	2	Left frontal	Not deleted	Mut	ND	Mut	Met	NMut	88.01	88.01
80	M	53	Anaplastic Astrocytoma	3	Right insula	ND	ND	Mut	NMut	Met	NMut	12.66	66.01
81	M	25	Anaplastic Astrocytoma	3	Right frontal	ND	Mut	Mut	Mut	Met	NMut	NA	NA
82	M	40	Anaplastic Astrocytoma	3	Left frontal	Not deleed	Mut	Mut	Mut	Met	NMut	77.68	77.68
83	M	32	Diffuse Astrocytoma	2	Left temporal	ND	Mut	Mut	Mut	Met	NMut	23.32	75.41
84	M	33	Diffuse Astrocytoma	2	Left frontal	ND	Mut	Mut	Mut	Met	NMut	44.16	44.16
85	M	36	Diffuse Astrocytoma	2	Left frontal	ND	Mut	Mut	Mut	Met	NMut	61.58	60.55
86	F	5	Non-tumor control (focal cortical dysplasia)	NA	Right frontal	NA	NA	NA	NA	NA	NA	NA	NA
87	F	2	Non-tumor control (focal cortical dysplasia)	NA	Right frontal	NA	NA	NA	NA	NA	NA	NA	NA
88	F	8	Non-tumor control (focal cortical dysplasia)	NA	Right temporal	NA	NA	NA	NA	NA	NA	NA	NA

Abbreviations: F: Female; M: Male; NA: Not applicable; ND: No data; Mut: Mutated; NMut: No mutated; Met: Methylated; NMet: No methylated.
